# Host range, morphological and genomic characterisation of bacteriophages with activity against clinical *Streptococcus agalactiae* isolates

**DOI:** 10.1371/journal.pone.0235002

**Published:** 2020-06-23

**Authors:** Lucy L. Furfaro, Matthew S. Payne, Barbara J. Chang

**Affiliations:** 1 The School of Medicine, Division of Obstetrics and Gynaecology, The University of Western Australia, Crawley, Australia; 2 The School of Biomedical Sciences, The Marshall Centre for Infectious Diseases Research and Training, The University of Western Australia, Crawley, Australia; Universidade de Lisboa Faculdade de Medicina, PORTUGAL

## Abstract

*Streptococcus agalactiae* or Group B Streptococcus (GBS) is a leading cause of sepsis in neonates. As a preventative measure prophylactic antibiotic administration is common in pregnant women colonised with GBS, but antibiotic-resistance and adverse effects on neonatal microbiomes may result. Use of bacteriophages (phages) is one option for targeted therapy. To this end, four phages (LF1 –LF4) were isolated from wastewater. They displayed lytic activity *in vitro* against *S*. *agalactiae* isolates collected from pregnant women and neonates, with 190/246 isolates (77.2%) and 10/10 (100%) isolates susceptible to at least one phage, respectively. Phage genomes ranged from 32,205–44,768 bp and all phages were members of the *Siphoviridae* family. High nucleotide identity (99.9%) was observed between LF1 and LF4, which were closely related to a putative prophage of *S*. *agalactiae*. The genome organisation of LF2 differed, and it showed similarity to a different *S*. *agalactiae* prophage, while LF3 was more closely related to a *Streptococcus pyogenes* phage. Lysogenic gene presence (integrase, repressor and regulatory modules), was suggestive of temperate phages. In a therapeutic context, temperate phages are not ideal candidates, however, the broad host range activity of these phages observed on clinical isolates *in vitro* is promising for future therapeutic approaches including bioengineered phage or lysin applications.

## 1. Introduction

As a leading neonatal pathogen, *Streptococcus agalactiae* is an important consideration for prophylactic antenatal treatment. Transmission of this organism to neonates from a colonised mother has led to the implementation of universal screening programs in various countries worldwide [1]. These consist of either risk- or culture-based approaches and result in the administration of intrapartum antibiotics. Colonisation by *S*. *agalactiae* occurs in 10–30% of pregnant women and as such there is widespread use of antibiotics given this treatment approach [2]. While this strategy has been effective in reducing the disease burden of early onset sepsis [1], there are implications to consider. Antibiotic exposure *in utero* as a result of intrapartum prophylaxis has been reported to have an impact on the infant microbiome, with description of altered community structure, species richness and diversity in the infant gut [3], the long term impact of which is not well understood [4].

In addition to microbiome-related effects is the potential for resistance emergence among *S*. *agalactiae* isolates. Clindamycin and erythromycin resistance have been frequently reported in the literature [5, 6] with rates as high as 70.8%, which is of particular concern for those women with penicillin allergies who often receive clindamycin as an alternative. Tolerance to penicillin was described as far back as 1994 by Betriu and colleagues [7] and since then further studies have observed reduced susceptibility to penicillin in *S*. *agalactiae* [8, 9]. Multi-drug resistance within a clone exhibiting intermediate susceptibility to penicillin has increased rapidly over the last decade in Japan [9]. This incidence of rising resistance coupled with the unknown effects of antenatal antibiotic administration on the infant are justified reasons for evaluation of alternative strategies for *S*. *agalactiae* prophylaxis during pregnancy.

Bacteriophages or phages, are being re-considered in the Western world as an alternative antimicrobial therapy in the face of increasing rates of antibiotic resistance [10]. These bacterial viruses typically have narrow antibacterial host ranges, are self-limiting, and are unable to infect human cells. These attributes make for an attractive solution to treatment of bacterial infections. Use of human phage therapy in the Western world has been a challenge and its use during pregnancy and the perinatal period presents an even greater one due to the vulnerability of this population [11]. Regardless, the prospects for phage therapy as a targeted alternative to antibiotics, particularly for the eradication of *S*. *agalactiae* in pregnant women, have huge potential. Antenatal *S*. *agalactiae* colonisation represents a niche opportunity in which a single bacterial species is the target; an ideal scenario considering the host specificity of phages. Phage therapy could potentially resolve issues associated with microbiome dysbiosis, as well as limiting the development of antibiotic resistance.

Several studies have explored phages active against *S*. *agalactiae*, however, none to date have isolated phages which are obligately lytic. Bai and colleagues isolated a total of four phages from bovine milk sources with the prospect of use in cases of bovine mastitis, of which *S*. *agalactiae* is an important cause [12, 13]. Domelier et al. isolated 36 phages by inducing 114 *S*. *agalactiae* isolates with mitomycin C and observing the resultant lytic activity [14]. Both studies failed to isolate phages that would be ideal for therapeutic use though, due to their temperate nature. With the risk of lysogeny in treated strains, other studies have focussed on utilising phage components such as lysins, which are responsible for bacterial cell wall degradation, as treatment options. One example of this is seen in a study by Cheng et al., which found that vaginal inoculation with *S*. *agalactiae* phage lysin PlyGBS (purified from a temperate *S*. *agalactiae* phage) successfully eradicated the colonising *S*. *agalactiae* isolates in a mouse model [15]. Our study aimed to isolate phages with lytic activity against clinical *S*. *agalactiae* isolates collected from pregnant women and neonates for potential use in future therapeutic ventures.

## 2. Materials and methods

### 2.1. Bacteriophage isolation

Wastewater from the Subiaco area, Western Australia was collected as an environmental source of phages (approval granted by Water Corporation, 39 Lemnos St, Shenton Park, Western Australia, Australia) and enriched using a panel of 20 *S*. *agalactiae* isolates (courtesy of Dr Kong and Prof. Gilbert, S1 Table) as per standard enrichment protocols [16]. The resulting 0.2 μm filtrate was spot tested on a range of *S*. *agalactiae* isolates to detect lytic phage activity [16]. Trypticase Soy Broth (Becton Dickinson, USA) supplemented with 12 mM Ca^2+^ was used in the enrichment process and as semi-solid media supplemented with 0.5% agar, while Tryptic Soy Agar (Becton Dickinson, USA) was used as solid media for whole plate and drop-on-plate (spot test) assays [17, 18]. Plaques were selected and purified on the propagating host in three separate purification rounds. The resulting pure phage suspensions were propagated to a high titre for further analysis.

### 2.2. Host range testing

A total of 256 clinical isolates were used for host range testing, including 246 carriage *S*. *agalactiae* isolates from consenting Western Australian (WA) pregnant women which were collected from 2015–2017 as part of a larger cohort study, Predict1000, which was approved by the Women and Newborn Health Service Human Research Ethics Committee (201535EW). In addition, 10 neonatal invasive disease *S*. *agalactiae* isolates from WA collected from 2012–2014 (courtesy of Dr Anthony Keil) were included. Other organisms that were included to test phage specificity were the closely-related species (n = 17), *Streptococcus pyogenes* (ATCC 12203), *Streptococcus equinus* (ATCC 15351), *Streptococcus mitis* (M2611), *Streptococcus dysgalactiae* subspecies *equisimilis* (NCTC 5371), *Streptococcus dysgalactiae* (clinical isolate), *Streptococcus gordonii* (ATCC 10558), *Streptococcus anginosus* (NCTC 8037), *Streptococcus salivarius* (clinical isolate), *Staphylococcus aureus* (ATCC 9144), *Staphylococcus saprophyticus* (ATCC 15305), *Staphylococcus epidermidis* (ATCC 14990), *Enterococcus faecalis* (ATCC 19433), *Lactobacillus crispatus* (BEI HM-637), *Lactobacillus gasseri* (BEI HM-104), *Lactobacillus jensenii* (BEI HM-105), *Lactobacillus johnsonii* (BEI HM-643) and *Lactobacillus rhamnosus* (BEI HM-106). All these Lactobacilli apart from *L*. *rhamnosus* are common vaginal bacteria associated with vaginal health: important for assessment of phage host range in the context of *S*. *agalactiae* antenatal prophylaxis. Host range testing involved the whole plate and drop-on-plate assays (including 1:10 phage dilution series), as described previously [17, 18]. Results were determined as positive for activity if the drop zone produced full clearing and/or plaques, and turbidity of the drop sites was noted. No observable activity on the lawn was recorded as negative for activity. Those with observed activity were assayed as a dilution series via drop-on-plate assay to detect plaques.

### 2.3. Clinical *S*. *agalactiae* host characteristics

All of the carriage *S*. *agalactiae* isolates were serotyped via multiplex assay to detect serotypes Ia, Ib and III [19] and remaining serotypes were determined by standard PCR and gel electrophoresis [20]. In addition to serotyping, 42 of the 246 carriage isolates, 10 of the 10 neonatal invasive isolates and 14 of the 15 reference isolates had whole genome sequence data available [21].

### 2.4. Microscopy

Transmission electron microscopy (TEM) was used to confirm phage presence and determine structure and taxonomy. Concentrated phage filtrates (10^10^ PFU/mL) were stained using 2% uranyl acetate on carbon-coated 200 mesh copper grids (SPI Supplies^®^, USA) and examined using a JEOL 2100 microscope operating at 120 kV. All TEM images collected were analysed using Fiji and Image J, including measurements of phage virions (minimum of ten single virions measured per phage) [22].

### 2.5. Sequencing

All phage lysates were treated with Turbo^™^ DNase (Life Technologies, USA) to remove bacterial host DNA contamination in the library preparation and phage DNA was extracted using the Phage DNA Isolation Kit (Norgen Biotek Corp., Canada). Extracted DNA was quantified using a Qubit^™^ fluorometer and 1.0 ng of DNA was used in the Illumina Nextera XT library preparation protocol as per manufacturer’s instructions (Illumina). Briefly, the genomic DNA was fragmented, indexed by PCR and purified using AMPure XP beads. Library pooling was performed after quantification of the library size using the LabChip^®^ GXII. The pooled DNA library was sequenced on an Illumina Nextseq and paired-end 300 bp reads generated. Genome analysis was carried out using Geneious (version 10.2.4) with phage genomes assembled using SPAdes (version 3.10.3) [23] and assessed for circularised genomes using Unicycler [24]. Open reading frames (ORFs) were predicted using Geneious based on Glimmer (version 3) [25] and the GenBank database was used to annotate these ORFs. RASTtk was also used for annotation [26]. Easyfig was used to produce genome maps and alignments [27] and Interactive Tree of Life (iTOL) was used to visualise the phylogenetic tree including 263 complete Streptococcus phage genomes, from the NCBI database, used for comparison [28]. PHASTER was used to evaluate the presence of putative prophages in reference isolates for comparison [29]. The genome sequences were deposited under the NCBI BioProject PRJNA631803 and GenBank under accession numbers MH853355 (LF1), MH853356 (LF2), MH853357 (LF3) and MH853358 (LF4).

## 3. Results

### 3.1. Phage isolation

**Fig 1:**
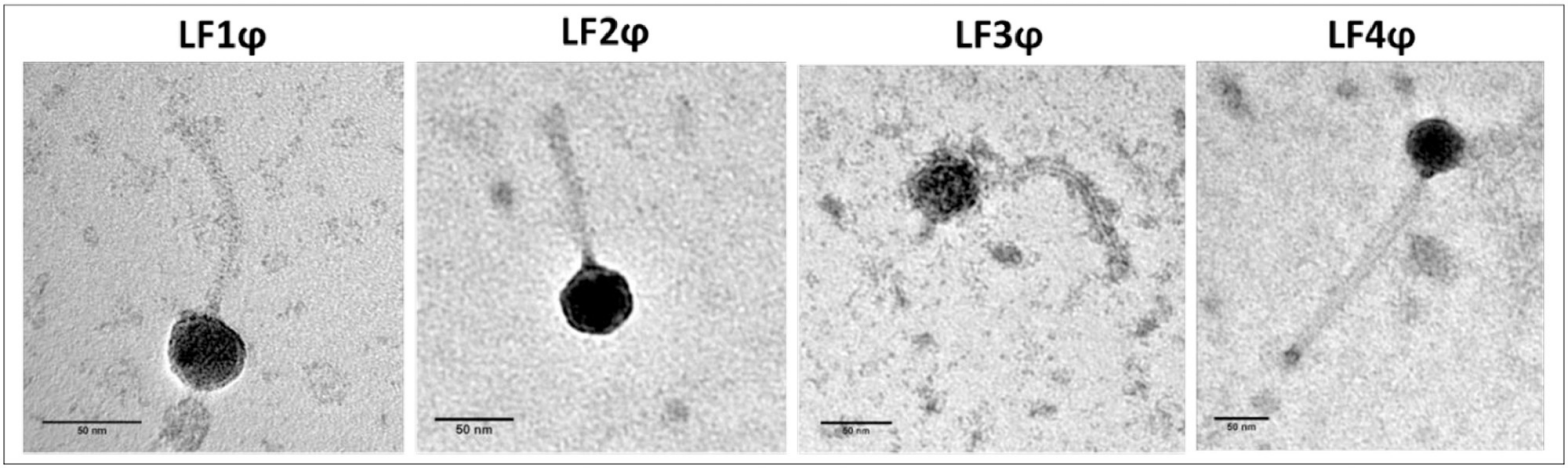
Transmission electron micrographs of the four *S*. *agalactiae* phages (LF1 –LF4). Scale bar of 50 nm included in each micrograph.

Four bacteriophages, LF1, LF2, LF3 and LF4, were isolated from local wastewater sources (Subiaco area, Western Australia, Australia) with propagating hosts of *S*. *agalactiae* reference isolates Kong_7 (LF1 and LF2), Kong_10 (LF3) and Kong_28 (LF4). All phages were found to belong to the *Siphoviridae* family based on TEM (Fig 1). Members of this family have an icosahedral head and long non-contractile tail; all phage dimensions were significantly different (p < 0.01) in at least one virion measurement according to student t-test (Table 1).

**Table 1:** *S*. *agalactiae* phages LF1, LF2, LF3 and LF4 virion measurements*.

Mean (nm) ± SD	LF1	LF2	LF3	LF4
**Tail length**	109.6 ± 2.9	108.6 ± 4.0	125.3 ± 4.6	198.9 ± 9.1
**Capsid width**	41.4 ± 1.5	43.3 ± 1.8	44.1 ± 4.1	46.3 ± 2.6
**Capsid length**	38.7 ± 2.2	42.9 ± 1.2	44 ± 4.2	45.7 ± 3.5

*A minimum of ten virions were measured per phage.

### 3.2. Host range activity

**Fig 2:**
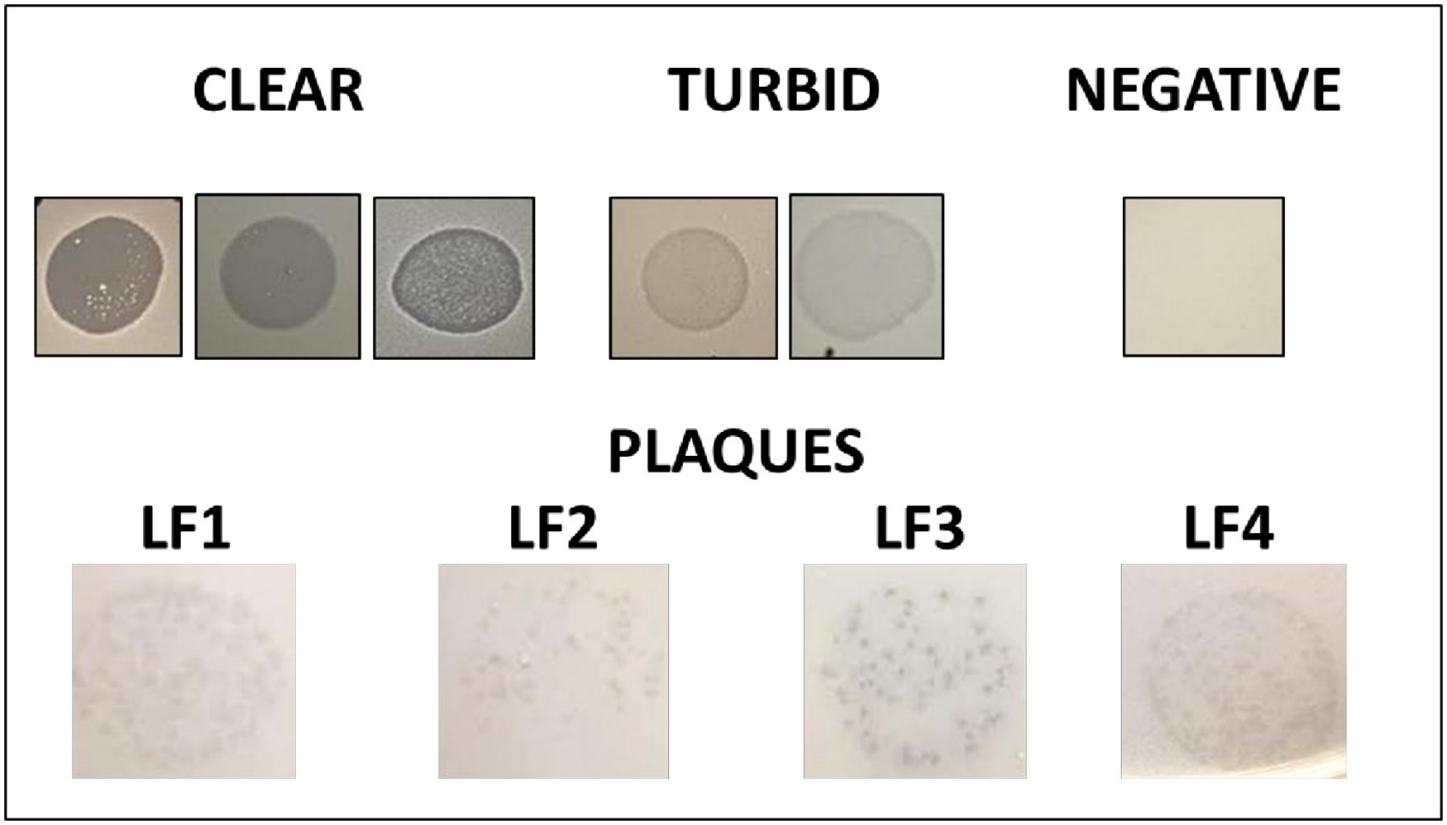
Representative examples of the spot test outcomes including plaques, clear, turbid and negative.

**Fig 3:**
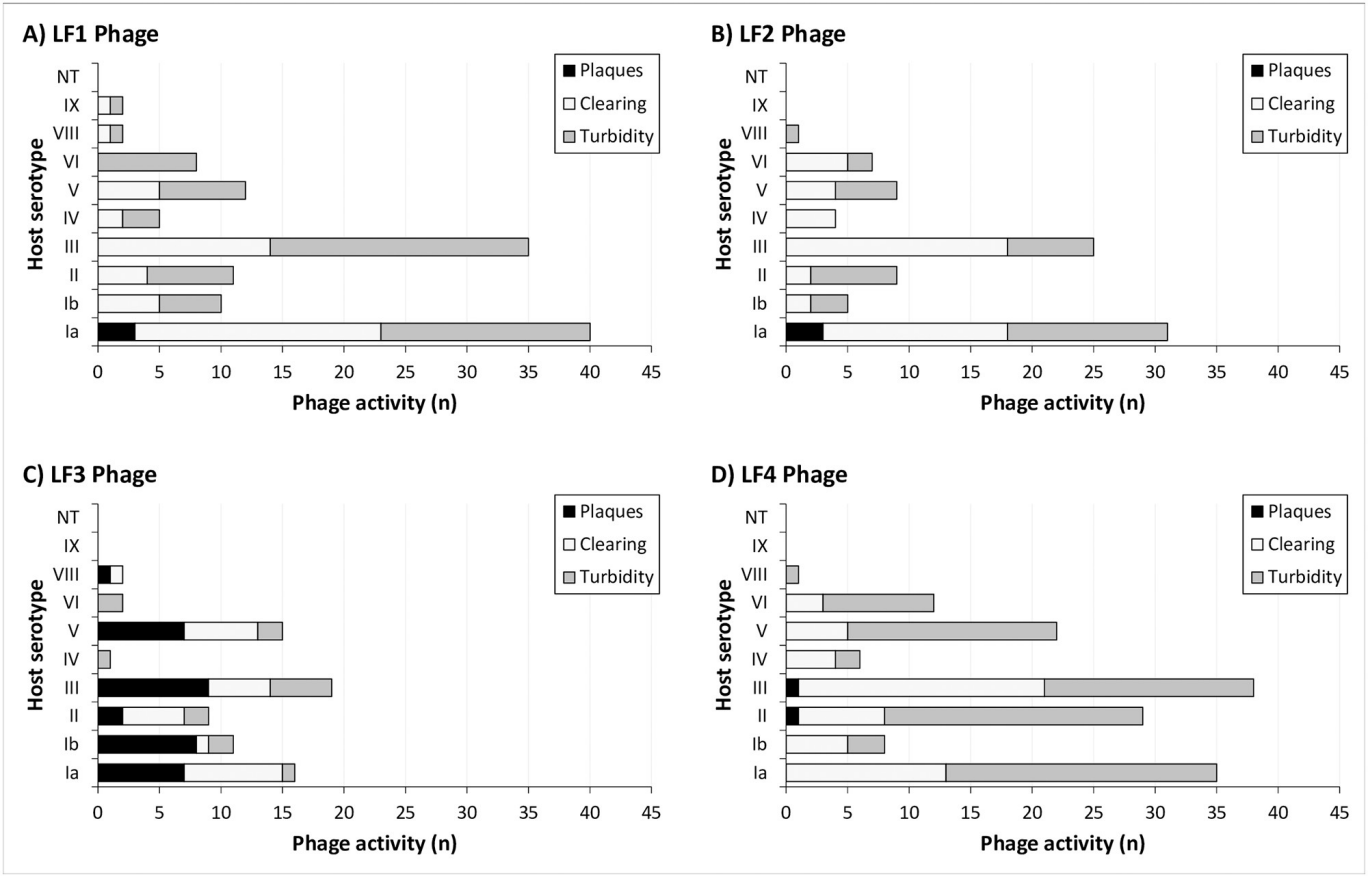
The relationship between *S*. *agalactiae* host serotype and LF1 (panel A, n = 125/256), LF2 (panel B, n = 91/256), LF3 (panel C, n = 75/256) and LF4 (panel D, n = 151/256) phage activity presented as the number of isolates that were sensitive (plaques, black; clearing, light grey; and turbidity, dark grey) and their serotype.

Activity of each phage against the 256 clinical *S*. *agalactiae* isolates (carriage and invasive) varied with 55 (21.5%), 53 (20.7%), 60 (23.5%) and 59 (23.1%) isolates sensitive (plaques or clearing) to LF1, LF2, LF3 and LF4, respectively (Table 2). Each phage had a different host range pattern. Between all four phages, activity was observed across 201 (78.2%) of the isolates, however, this consisted of both clear and turbid clearing results (Fig 2). The specificity testing showed no activity of the phages against closely-related organisms or commensal vaginal bacteria tested, except for the single clinical *S*. *salivarius* isolate that was sensitive to LF1, LF2 and LF4 phages. Serotyping of the 256 clinical *S*. *agalactiae* isolates revealed the presence of serotypes Ia, Ib, II, III, IV, V, VI, VIII, IX and non-typeable (NT) strains. Comparison between individual phage activities based on serotype suggested that activity was serotype independent (Fig 3).

**Table 2:** The host range activity of four *S*. *agalactiae* phages against clinical carriage (antenatal) and disease (neonatal) *S*. *agalactiae* isolates (n = 256). The activity is divided into plaque formation, clear lysis zones, turbid zones and no activity.

	Phage activity against GBS isolates n (%)			
	Plaques	Lysis zone	Turbid	Negative
**LF1**	3 (1.2)	52 (20.3)	70 (27.3)	131 (51.2)
**LF2**	3 (1.2)	50 (19.5)	38 (14.8)	165 (64.5)
**LF3**	34 (13.3)	26 (10.2)	15 (5.9)	181 (70.7)
**LF4**	2 (0.8)	57 (22.3)	92 (35.9)	105 (41.0)

### 3.3. Phage genomic analysis

**Fig 4:**
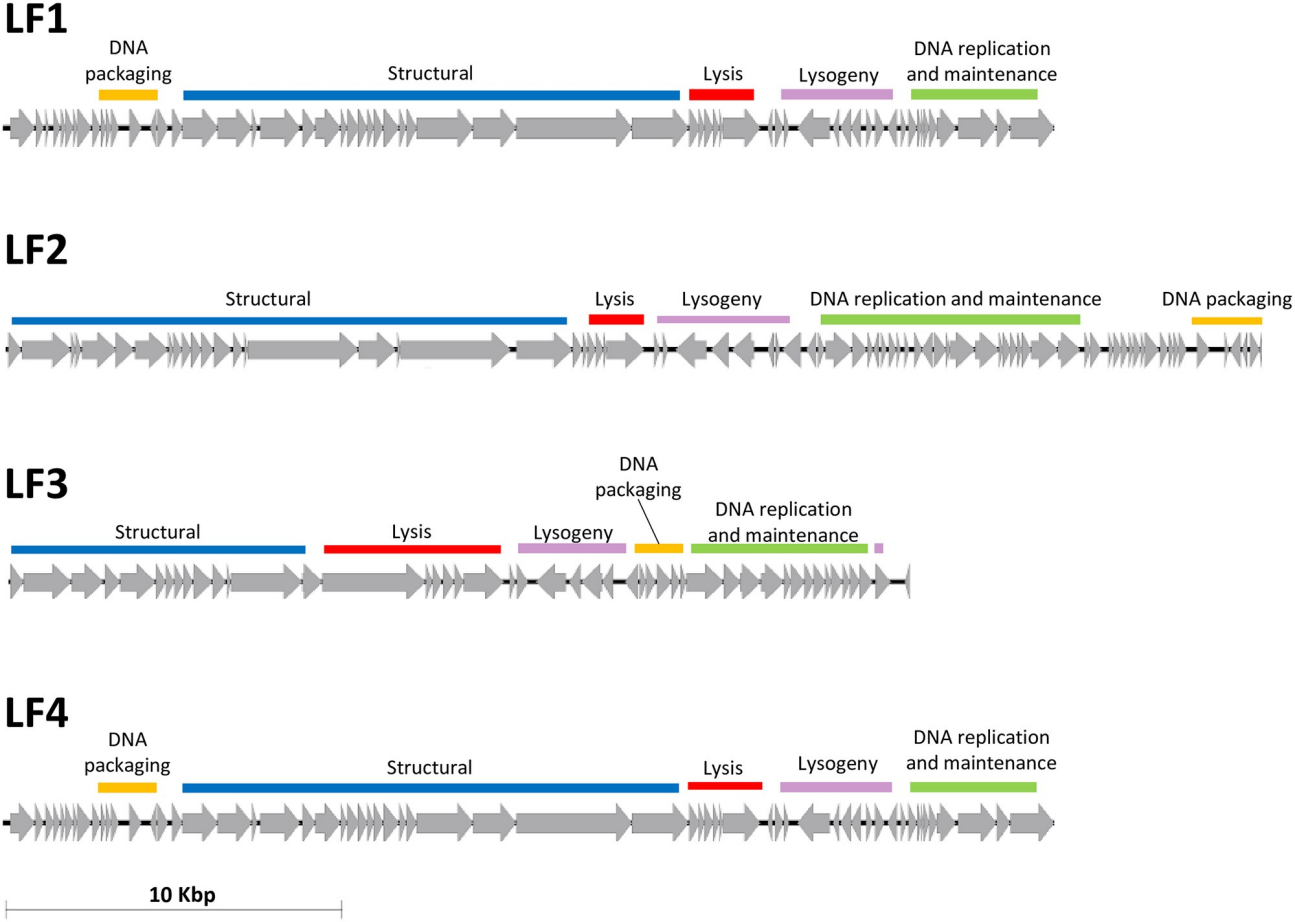
The organisation of genes in the genomes of four *S*. *agalactiae* phages with modules highlighted.

**Fig 5:**
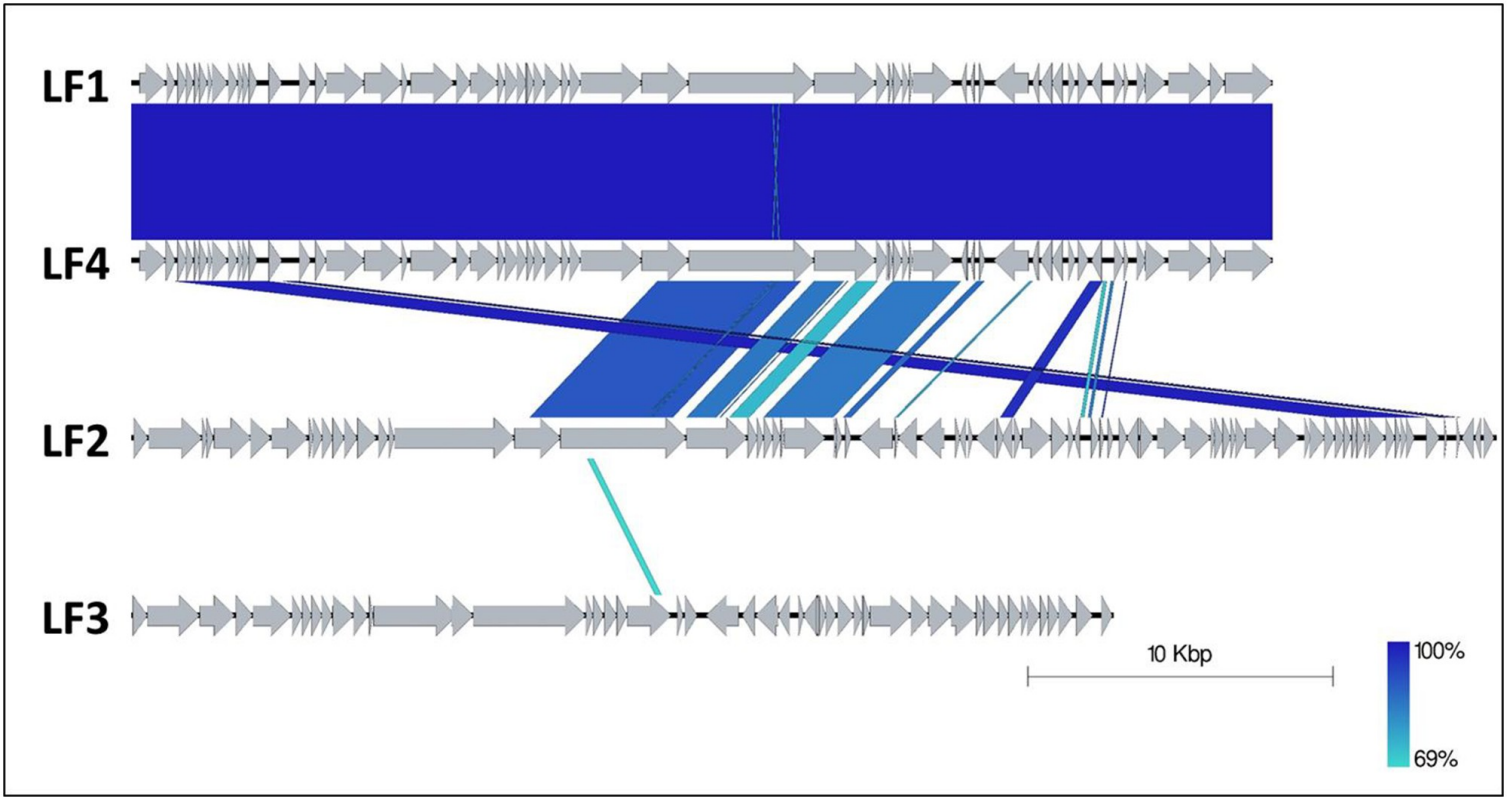
Comparative alignment of all *S*. *agalactiae* phages demonstrating the percentage nucleotide similarity of the different genomes (colour scale percentage similarity).

Assembly of the phage sequence data showed variation in size and G/C% across three of the four phages. LF1 and LF4 were highly similar, and assembly resulted in complete circularised genomes for each (total length 37,421 bp) with G/C content of 37% and read coverage of 14,862 x and 16,724 x, respectively. LF2 (44,768 bp) and LF3 (32,205 bp) were annotated and assessed based on their largest contig, which represented all gene modules expected in a phage genome and contained 36.7% and 40.2% G/C content and read coverage of 5,693 x and 24,143 x, respectively. LF2 and LF3 are considered partial genomes as the DNA ends have not been confirmed. A total of 65, 87, 55 and 65 open reading frames (ORFs) were predicted by Glimmer and 60, 76, 47 and 60 ORFs were predicted by RASTtk for phages LF1, LF2, LF3 and LF4, respectively. Annotation using BLASTN and RASTtk revealed similar structural arrangement of gene modules for LF1, LF2 and LF4, however LF3 was distinct (Fig 4). All phages were aligned to assess their sequence similarity (Fig 5). LF1 and LF4 showed 99.9% identity (52 bp difference overall). LF2 shared regions of similarity with LF1 and LF4, however, LF3 showed the least similarity, sharing only a small section within the structural module with LF2. BLAST analysis of LF1 and LF4 suggested similarity to putative prophages of *S*. *agalactiae* A909 (Accession#: CP000114; S1 Fig) which had no previous function characterised according to PHASTER. LF2 appeared to have regions of similarity with the putative prophage characterised for *S*. *agalactiae* BM110 (Accession#: LT714196; S1 Fig), but rearranged. Lastly, LF3 shared similarity with a partial region of the putative prophage from *S*. *agalactiae* SG-M6 (Accession#: CP021869.1; S1 Fig). These putative prophages have not been functionally described previously and our genome data do not cover the entire putative prophages.

### 3.4. Annotations

Numerous methods were assessed to annotate the phage genomes including RASTtk (customised to phage annotations), Glimmer and BLAST (S2 Table). This analysis revealed key genes involved in lysogeny including those encoding integrase, excisionase, Cro/CI repressors. A toxin-associated gene, annotated as paratox, was found adjacent to the integrase gene in LF1 and LF4 and near transcriptional regulator genes in LF2. LF3 did not contain paratox, however, BLASTN analysis revealed 100% identity to a toxin-antitoxin system in *S*. *pyogenes* Str03 phage (Accession#: KY363359) and high similarity to an additional antitoxin HicB protein family gene (~98% identity). No toxin genes were identified in the LF phages. A tyrosine tRNA (tRNA-Tyr-ATA) was identified within LF2 by RASTtk, located at the terminus of the genome. Integrase genes of all LF phages were reported as tyrosine recombinases by HHPred, with <43% similarity except for LF1 and LF4 which shared 99.7%. All putative lysins of our four phages were tested using BLASTP to assess novelty. LF1, LF2 and LF4 shared similarity with the Cpl-1 lysozyme/muramidase. LF1 and LF4 shared 98.6% (six amino acid differences) and 99.8% (one amino acid difference) identity with Streptococcus phage B30 peptidoglycan endolysin protein (AAN28166.2), respectively. LF2 shared 99.8% similarity with *Streptococcus agalactiae* lysin protein (WP_025197090.1) and 93% identity to the LF1 and LF4 endolysin protein. Distinct from these phages with only 12% identity to the other lysins, LF3 was found to share 100% identity to *S*. *agalactiae* glucosaminidase domain-containing protein (WP_000512610.1).

### 3.5. Phylogenetic analysis

**Fig 6:**
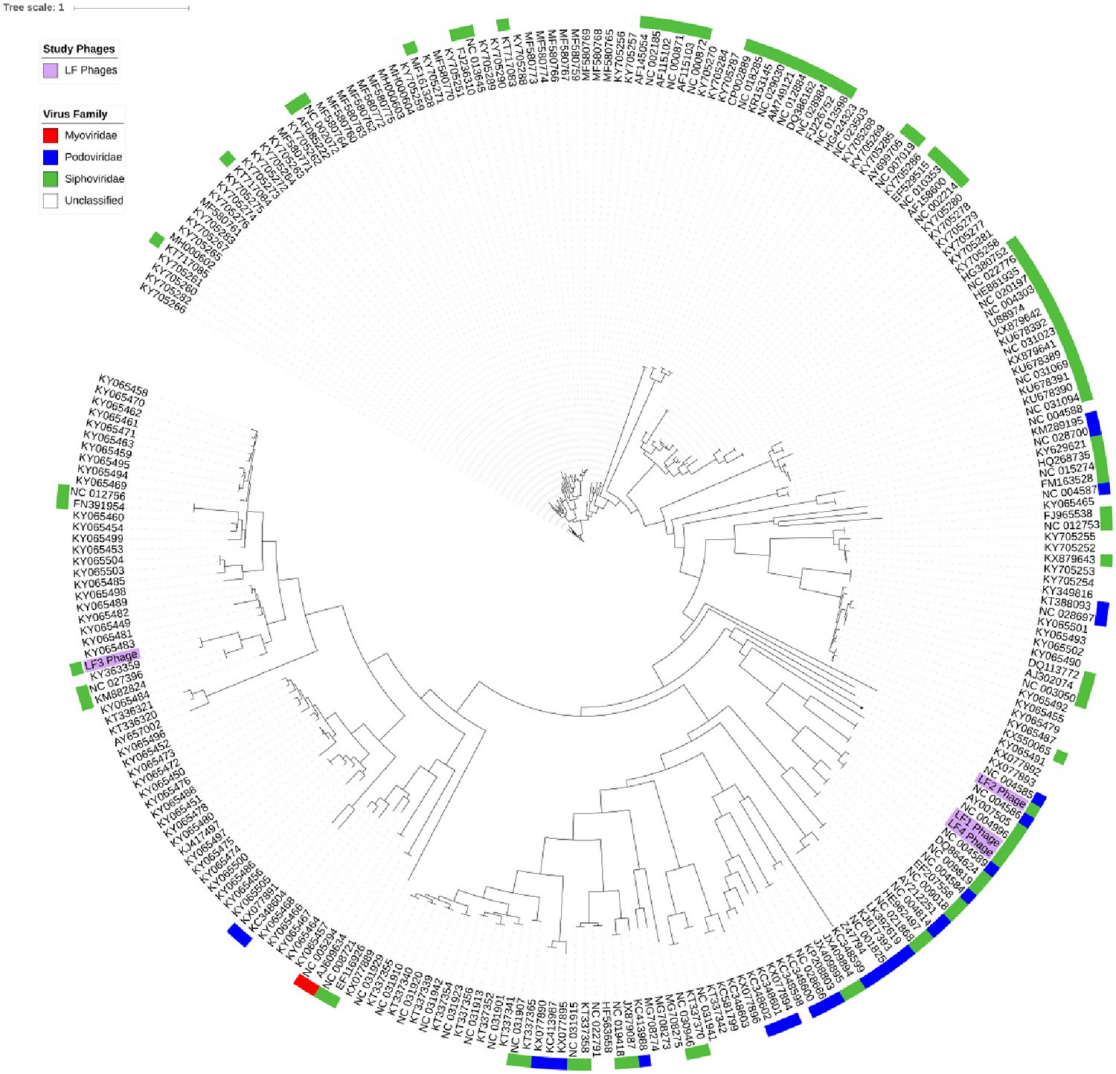
The neighbour-joining phylogenetic tree analysis of LF1 –LF4 phages (purple) and 263 complete Streptococcus phage genomes and their respective viral families.

The four LF phages in the context of complete Streptococcus phage genomes shared <60% similarity with the closest ancestor of the 263 Streptococcus phages assessed (Fig 6). The distances between the LF phages within the neighbour-joining tree mirrored the percentage similarity observed in Fig 5, with LF1 and LF4 highly similar, LF2 somewhat similar and LF3 distantly related. Alignment of LF1 and LF4, LF2 and LF3 with neighbouring Streptococcus phages observed a 41.4%, 41.3% and 93% similarity, respectively (S2 Fig). The Streptococcus phage tree included the virus family where available, and was predominated by *Siphoviridae*, however, the closest ancestors to LF2 were *Podoviridae* family members.

## 4. Discussion

We describe four temperate *S*. *agalactiae* phages isolated from wastewater with activity against clinical *S*. *agalactiae* human isolates. Transmission electron micrographs revealed all phages were from the *Siphoviridae* family. A broad range of host activity was observed of all phages against both antenatal carriage and neonatal disease *S*. *agalactiae* isolates, individually spanning multiple serotypes. While host range overlapped, each phage had a different host range pattern including unique activity of LF1, LF2, LF3 and LF4 against 8, 3, 20 and 25 *S*. *agalactiae* isolates screened, respectively. These differences further indicated that the four phages were distinct. It should be noted that the spot test, however, has limitations because of the instances of false positives [30]. The detection of plaques via dilution screening was able to provide confidence that activity was due to phages and not bacteriocins.

Phylogenetic comparison with other completed Streptococcus phage genomes showed clustering which was aligned with other phages on neighbouring branches (S2 Fig). Whole genome analysis revealed a high sequence similarity between LF1 and LF4 suggesting these were highly related with only 52 bp (43 amino acid) differences overall. This similarity contrasts with the differences observed phenotypically and structurally. These phages shared 108 isolates as hosts, but LF4 was active against an additional 50 isolates, while LF1 lysed only an additional 24 isolates. Structurally the LF4 tail measured almost twice the length of that of LF1, and its capsid was significantly larger, the latter possibly related to two amino acid differences in the major capsid protein (99% similarity to WP_0008410241.1). The amino acid differences between the two phages were seen across numerous conserved hypothetical proteins in addition to terminase, lysin, capsid and other structural proteins, including a minor structural protein within the tail protein cluster (ORF32). This ORF is the longest gene in the genome and may represent the tape-measure protein, however, no annotations have revealed a direct similarity to other tape-measure proteins. Several tail proteins contained amino acid differences between LF1 and LF4 phages, however, the direct cause of the morphological difference has not been confirmed. Previous analyses of mutations in the structural proteins and tail fibres that affect host range were reported by Jacobs-Sera and colleagues [31]. They observed single point mutations resulting in an amino acid substitution in the putative minor tail protein genes of mycobacteriophages and attributed the differing host ranges to these changes [31]. Similarly, we found a single amino acid substitution in the ORF32 protein of LF1 and LF4 phages in addition to observable host range differences. ORF32 aligns (31% identity, 98% cover, E value 2e^-47^) with the genes present in several *S*. *thermophilus* phages encoding antireceptor proteins responsible for host specificity [32].

The lysogenic regions including integrase and repressor genes detected within our sequence data are suggestive that all four phages are temperate. This corresponds with the similarity with partial regions of *S*. *agalactiae* whole genomes reported by BLAST. LF1 and LF4 results were indicative of a prophage from *S*. *agalactiae* A909, a well-known reference isolate, however, the regions of phage coverage did not correspond with the putative phages outlined by PHASTER. Similarly, LF2 contained regions of similarity to *S*. *agalactiae* BM110: although rearranged gene modules are evident this is not uncommon in phages [33]. LF3 also shared similarity with *S*. *agalactiae* SG-M6, which corresponded with a partial region of this putative prophage. Synteny differed between LF3 and the other phages, but LF3 genome organisation resembled that of other temperate *S*. *agalactiae* phages reported including JX01 [13]. These observed similarities with putative prophages indicate that *S*. *agalactiae* isolates are the origin of the four phages, which have presumably been released as virions into the environment by spontaneous induction from their hosts. Additional experiments are required to functionally confirm these findings which include determining whether lysogens can be formed *in vitro* and assessing the conditions of prophage induction.

Domelier and colleagues reported the activity of chemically-induced phages originating from human *S*. *agalactiae* isolates. They observed a lineage-based specificity whereby phages induced from all clonal complexes (CC), excluding CC17, only lysed isolates of a similar evolutionary origin. In contrast, phages induced from CC17 *S*. *agalactiae* isolates demonstrated lytic activity against all other lineages except for CC23 [14]. The range of serotypes sensitive to each of the LF phages does not support this finding, unless all four phages originated from a CC17 strain, phages from which Domelier and others showed had a broader host range. Further to this point, Salloum et al. revealed the clustering of prophage DNA into five groups within a cohort of 142 *S*. *agalactiae* isolates that caused meningitis and bacteraemia in adults and neonates. They showed differences in prophage DNA content between serotypes, STs and CCs and concluded with the suggestion that mechanisms of gene transfer from cell to cell mediated by phages are specific to each intraspecies lineage [34]. This is an interesting notion, given that we have evaluated the serotype and ST for a proportion of the isolates used in our host range studies, and found to the contrary, that phage activity was observed across all serotypes and STs. While the majority of these isolates are maternal carriage isolates, the few neonatal invasive disease isolates (n = 10) were also similar with regard to this variation. This activity would suggest that the polysaccharide capsules containing these serotype determinants are not responsible for phage attachment, as this is often a very specific binding, and may be a result of phage evolutionary adaptation to the infection of different serotypes.

LF2 contained a tRNA gene which was not observed in the other three phage genomes. Genes encoding tRNAs are common in temperate phage integration sites: in this regard, a serine tRNA gene was reported to be the integration site for *S*. *pyogenes* phage T12 with a 96 bp homologous sequence in the phage attachment site *attP* corresponding to the 3’ end of the bacterial tRNA gene [35], while a serine tRNA gene was annotated in *S*. *agalactiae* JX01 phage genome by Bai et al. [13]. This interesting link to phage integration may relate to potential lysogeny by LF2. On the other hand, presence of tRNA genes in phage genomes has often been attributed to the promotion of more efficient translation of phage-derived mRNA [36].

When considered for therapeutic use, individual phages are not likely to be used, rather a cocktail of different phages to cover an extensive host range [10]. A cocktail of the phages isolated from this study would result in coverage of 50.1% as a conservative estimate, and as many as 77.3% when including turbid zones, of the clinical *S*. *agalactiae* isolates screened. Given the possible temperate nature of these phages they are not recommended candidates for phage therapy, however, as no obligately lytic phages have been described with activity against *S*. *agalactiae*, temperate phages may yet be an option. A similar scenario is observed for *Clostridioides difficile*, in which to date only temperate phages have been isolated, yet these have been pursued as a therapeutic option regardless of their potential disadvantages, which has resulted in promising results *in vitro* [37]. The activity observed makes for a case to pursue the isolation and characterisation of *S*. *agalactiae* phages and indicates the potential for future bioengineering of those described here.

The isolation and host range, morphological and genomic characterisation of the four LF phages adds to our knowledge of *S*. *agalactiae* phages and highlights the activity of temperate phages on relevant clinical isolates comprising both antenatal colonising isolates and neonatal invasive disease isolates. Further characterisation is required to confirm the temperate nature of these phages and will be an important step for assessment of bioengineering potential of these phages in future studies.

## Supporting information

S1 Fig Comparative alignment of LF1 (MH853355)/LF4 (MH853358), LF2 (MH853356) and LF3 (MH853357) *S*. *agalactiae* phages with putative prophages from isolates *S*. *agalactiae* A909 (CP000114), BM110 (LT714196) and SG-M6 (CP021869), respectively. A scale bar representing 10 Kb is included for each alignment in addition to a colour scale demonstrating the percentage nucleotide similarity of the different genomes.
(PDF)

S2 Fig Comparative alignment of LF1 (MH853355)/LF4 (MH853358), LF2 (MH853356) and LF3 (MH853357) *S*. *agalactiae* phages with closely-related Streptococcal phages SM1 (NC_004996), 315.2 (NC_004585) and Str03 (KY363359) respectively. A scale bar representing 10 Kb is included for each alignment in addition to a colour scale demonstrating the percentage nucleotide similarity of the different genomes.
(PDF)

S1 Table Details of the isolates used in the current study. Details include source, capsular genotype (CPS), sequence type (ST), link to whole genome sequence (WGS) data and LF phage activity. Those isolates used as the enrichment panel are denoted with an asterisk (*).
(PDF)

S2 Table Genome annotations of *S*. *agalactiae phages* LF1- LF4. (PDF)
