# Supplementary material for: Host range, morphological and genomic characterisation of bacteriophages with activity against clinical Streptococcus agalactiae isolates
Source: PLoS One. 2020 Jun 23;15(6):e0235002. doi: 10.1371/journal.pone.0235002 (PMC7310703; doi:10.1371/journal.pone.0235002)
Supplement: S1 Fig — A scale bar representing 10 Kb is included for each alignment in addition to a colour scale demonstrating the percentage nucleotide similarity of the different genomes. (PDF) [file pone.0235002.s001.pdf]

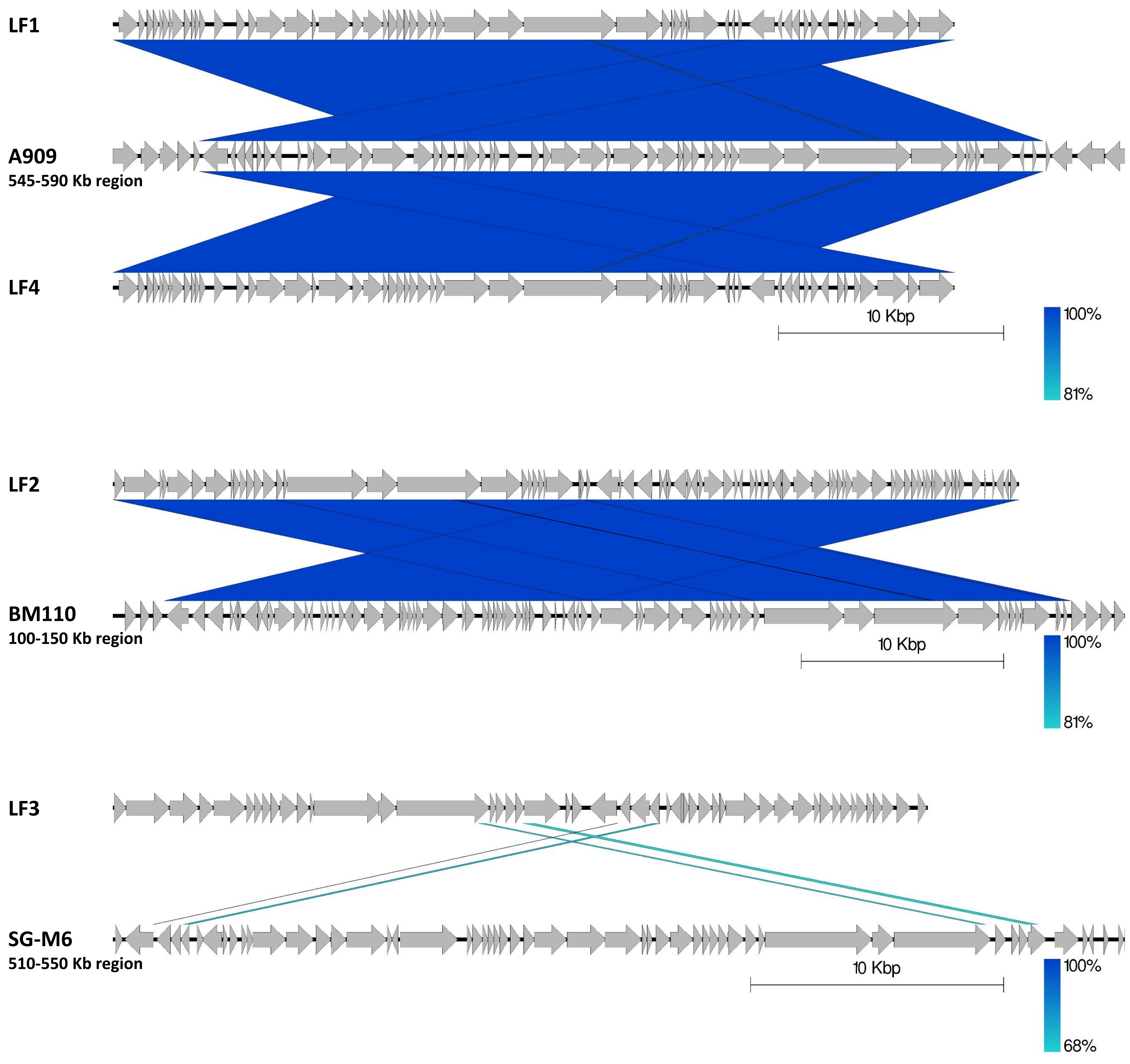

S1 Fig. Comparative alignment of LF1 (MH853355)/LF4 (MH853358), LF2 (MH853356) and LF3 (MH853357) *S. agalactiae* phages with putative prophages from isolates *S. agalactiae* A909 (CP000114), BM110 (LT714196) and SG-M6 (CP021869), respectively. A scale bar representing 10 Kb is included for each alignment in addition to a colour scale demonstrating the percentage nucleotide similarity of the different genomes.
