# Supplementary material for: Host range, morphological and genomic characterisation of bacteriophages with activity against clinical Streptococcus agalactiae isolates
Source: PLoS One. 2020 Jun 23;15(6):e0235002. doi: 10.1371/journal.pone.0235002 (PMC7310703; doi:10.1371/journal.pone.0235002)
Supplement: S1 Table — Details include source, capsular genotype (CPS), sequence type (ST), link to whole genome sequence (WGS) data and LF phage activity. Those isolates used as the enrichment panel are denoted with an asterisk (*). (PDF) [file pone.0235002.s003.pdf]

S1 Table. Details of the isolates used in the current study. Details include source, capsular genotype (CPS), sequence type (ST), link to whole genome sequence (WGS) data and LF phage activity. Those isolates used as the enrichment panel are denoted with an asterisk (*).

Isolate	Source	CPS	WGS	ST	LF1 activity	LF2 activity	LF3 activity	LF4 activity
***Streptococcus agalactiae* isolates**								
**1V**	Carriage (vaginal swab)	**II**	N/A	N/A	NEG	CLEAR	NEG	TURBID
**1R**	Carriage (rectal swab)	**VI**	N/A	N/A	TURBID	CLEAR	NEG	CLEAR
**8V**	Carriage (vaginal swab)	**VI**	N/A	N/A	NEG	CLEAR	NEG	TURBID
**8R**	Carriage (rectal swab)	**VI**	N/A	N/A	NEG	CLEAR	NEG	CLEAR
**9R**	Carriage (rectal swab)	**VI**	N/A	N/A	NEG	CLEAR	NEG	CLEAR
**10V**	Carriage (vaginal swab)	**Ia**	N/A	N/A	TURBID	CLEAR	NEG	TURBID
**10R**	Carriage (rectal swab)	**Ia**	N/A	N/A	CLEAR	TURBID	NEG	CLEAR
**12V**	Carriage (vaginal swab)	**II**	N/A	N/A	NEG	TURBID	NEG	TURBID
**12R**	Carriage (rectal swab)	**II**	N/A	N/A	NEG	TURBID	NEG	TURBID
**13V**	Carriage (vaginal swab)	**V**	N/A	N/A	NEG	CLEAR	NEG	CLEAR
**13R**	Carriage (rectal swab)	**V**	N/A	N/A	NEG	TURBID	NEG	TURBID
**22V**	Carriage (vaginal swab)	**III**	PubMLST ID: 4487	17	CLEAR	CLEAR	NEG	CLEAR
**22R**	Carriage (rectal swab)	**III**	N/A	N/A	CLEAR	CLEAR	NEG	CLEAR
**23V**	Carriage (vaginal swab)	**IV**	PubMLST ID: 4488	196	TURBID	CLEAR	NEG	TURBID
**23R**	Carriage (rectal swab)	**IV**	N/A	N/A	CLEAR	CLEAR	NEG	CLEAR
**26V**	Carriage (vaginal swab)	**NT**	PubMLST ID: 4495	19	NEG	NEG	NEG	NEG
**26R**	Carriage (rectal swab)	**NT**	PubMLST ID: 4494	19	NEG	NEG	NEG	NEG
**35V**	Carriage (vaginal swab)	**Ia**	PubMLST ID: 4507	23	TURBID	TURBID	NEG	TURBID
**36V**	Carriage (vaginal swab)	**V**	N/A	N/A	NEG	NEG	NEG	NEG
**36R**	Carriage (rectal swab)	**V**	N/A	N/A	NEG	TURBID	NEG	TURBID
**40V**	Carriage (vaginal swab)	**V**	N/A	N/A	NEG	NEG	NEG	NEG
**40R**	Carriage (rectal swab)	**V**	N/A	N/A	CLEAR	NEG	NEG	CLEAR
**47V**	Carriage (vaginal swab)	**Ia**	PubMLST ID: 4515	23	TURBID	CLEAR	NEG	CLEAR
**48V**	Carriage (vaginal swab)	**Ia**	PubMLST ID: 4516	23	CLEAR	CLEAR	NEG	NEG
**48R**	Carriage (rectal swab)	**Ia**	N/A	N/A	CLEAR	CLEAR	NEG	CLEAR
**53V**	Carriage (vaginal swab)	**Ia**	N/A	N/A	NEG	NEG	NEG	NEG
**53R**	Carriage (rectal swab)	**Ia**	N/A	N/A	NEG	NEG	NEG	NEG
**61V**	Carriage (vaginal swab)	**II**	N/A	N/A	TURBID	CLEAR	NEG	CLEAR

**61R**	Carriage (rectal swab)	**II**	N/A	N/A	NEG	NEG	NEG	TURBID
**64V**	Carriage (vaginal swab)	**Ia**	N/A	N/A	CLEAR	CLEAR	NEG	CLEAR
**64R**	Carriage (rectal swab)	**Ia**	N/A	N/A	CLEAR	CLEAR	NEG	CLEAR
**69V**	Carriage (vaginal swab)	**Ia**	N/A	N/A	NEG	CLEAR	NEG	TURBID
**69R**	Carriage (rectal swab)	**Ia**	N/A	N/A	NEG	NEG	NEG	NEG
**84V**	Carriage (vaginal swab)	**III**	N/A	N/A	TURBID	NEG	PLAQUE	TURBID
**84R**	Carriage (rectal swab)	**III**	N/A	N/A	TURBID	CLEAR	NEG	CLEAR
**87V**	Carriage (vaginal swab)	**Ib**	N/A	N/A	TURBID	NEG	PLAQUE	TURBID
**87R**	Carriage (rectal swab)	**Ib**	N/A	N/A	NEG	NEG	PLAQUE	NEG
**90V**	Carriage (vaginal swab)	**III**	PubMLST ID: 4523	19	TURBID	CLEAR	NEG	CLEAR
**90R**	Carriage (rectal swab)	**III**	N/A	N/A	TURBID	CLEAR	NEG	CLEAR
**92V**	Carriage (vaginal swab)	**III**	PubMLST ID: 4524	17	NEG	NEG	NEG	TURBID
**92R**	Carriage (rectal swab)	**III**	N/A	N/A	NEG	NEG	NEG	CLEAR
**97V**	Carriage (vaginal swab)	**Ia**	N/A	N/A	NEG	NEG	NEG	TURBID
**97R**	Carriage (rectal swab)	**Ia**	N/A	N/A	NEG	NEG	NEG	TURBID
**104V**	Carriage (vaginal swab)	**Ib**	PubMLST ID: 4473	15	NEG	NEG	NEG	NEG
**104R**	Carriage (rectal swab)	**Ib**	N/A	N/A	NEG	NEG	NEG	NEG
**105R**	Carriage (rectal swab)	**II**	N/A	N/A	NEG	NEG	NEG	NEG
**107V**	Carriage (vaginal swab)	**III**	PubMLST ID: 4475	335	TURBID	CLEAR	NEG	CLEAR
**107R**	Carriage (rectal swab)	**III**	N/A	N/A	TURBID	CLEAR	NEG	CLEAR
**108V**	Carriage (vaginal swab)	**V**	N/A	N/A	NEG	TURBID	NEG	TURBID
**108R**	Carriage (rectal swab)	**III**	N/A	N/A	NEG	CLEAR	NEG	CLEAR
**110V**	Carriage (vaginal swab)	**III**	N/A	N/A	NEG	NEG	PLAQUE	NEG
**110R**	Carriage (rectal swab)	**III**	N/A	N/A	TURBID	NEG	PLAQUE	TURBID
**112V**	Carriage (vaginal swab)	**Ia**	PubMLST ID: 4477	23	TURBID	NEG	NEG	TURBID
**112R**	Carriage (rectal swab)	**Ia**	PubMLST ID: 4476	23	CLEAR	CLEAR	NEG	CLEAR
**121V**	Carriage (vaginal swab)	**Ia**	PubMLST ID: 4478	23	NEG	NEG	NEG	NEG
**123V**	Carriage (vaginal swab)	**Ia**	PubMLST ID: 4479	23	TURBID	NEG	NEG	TURBID
**132V**	Carriage (vaginal swab)	**III**	PubMLST ID: 4480	17	NEG	NEG	NEG	NEG
**132R**	Carriage (rectal swab)	**III**	N/A	N/A	CLEAR	CLEAR	NEG	CLEAR
**133V**	Carriage (vaginal swab)	**V**	N/A	N/A	TURBID	NEG	NEG	TURBID
**133R**	Carriage (rectal swab)	**V**	N/A	N/A	NEG	NEG	NEG	NEG
**137V**	Carriage (vaginal swab)	**III**	PubMLST ID: 4481	529	NEG	TURBID	CLEAR	TURBID

**137R**	Carriage (rectal swab)	**III**	N/A	N/A	NEG	NEG	NEG	TURBID
***152V**	Carriage (vaginal swab)	**Ia**	PubMLST ID: 4482	1437	**PLAQUE**	**PLAQUE**	NEG	**CLEAR**
**152R**	Carriage (rectal swab)	**Ia**	N/A	N/A	**CLEAR**	**CLEAR**	NEG	TURBID
**160V**	Carriage (vaginal swab)	**II**	PubMLST ID: 4483	12	NEG	NEG	NEG	NEG
**160R**	Carriage (rectal swab)	**II**	N/A	N/A	NEG	NEG	NEG	NEG
**162V**	Carriage (vaginal swab)	**V**	N/A	N/A	NEG	NEG	**CLEAR**	NEG
**162R**	Carriage (rectal swab)	**V**	N/A	N/A	NEG	NEG	**CLEAR**	NEG
**175R**	Carriage (rectal swab)	**Ia**	N/A	N/A	NEG	NEG	**PLAQUE**	NEG
**176V**	Carriage (vaginal swab)	**II**	N/A	N/A	NEG	NEG	NEG	TURBID
**176R**	Carriage (rectal swab)	**II**	N/A	N/A	NEG	NEG	NEG	TURBID
**183V**	Carriage (vaginal swab)	**Ib**	N/A	N/A	**CLEAR**	TURBID	NEG	**CLEAR**
**183R**	Carriage (rectal swab)	**Ib**	N/A	N/A	NEG	TURBID	NEG	NEG
**185V**	Carriage (vaginal swab)	**II**	N/A	N/A	NEG	NEG	NEG	TURBID
**185R**	Carriage (rectal swab)	**II**	N/A	N/A	NEG	NEG	NEG	NEG
**196V**	Carriage (vaginal swab)	**III**	N/A	N/A	NEG	NEG	NEG	NEG
**196R**	Carriage (rectal swab)	**V**	N/A	N/A	NEG	NEG	NEG	NEG
**198V**	Carriage (vaginal swab)	**III**	N/A	N/A	NEG	NEG	NEG	NEG
**200V**	Carriage (vaginal swab)	**Ia**	PubMLST ID: 4643	23	NEG	NEG	NEG	NEG
**200R**	Carriage (rectal swab)	**Ia**	N/A	N/A	NEG	NEG	NEG	NEG
**202V**	Carriage (vaginal swab)	**V**	PubMLST ID: 4485	41	NEG	NEG	**PLAQUE**	NEG
**202R**	Carriage (rectal swab)	**V**	N/A	N/A	**CLEAR**	**CLEAR**	NEG	**CLEAR**
**218V**	Carriage (vaginal swab)	**V**	PubMLST ID: 4486	1	NEG	NEG	NEG	NEG
**221V**	Carriage (vaginal swab)	**Ia**	N/A	N/A	NEG	NEG	NEG	NEG
**221R**	Carriage (rectal swab)	**Ia**	N/A	N/A	NEG	NEG	NEG	NEG
**226R**	Carriage (rectal swab)	**II**	N/A	N/A	NEG	NEG	**CLEAR**	NEG
**230R**	Carriage (rectal swab)	**VI**	N/A	N/A	NEG	NEG	NEG	NEG
**231V**	Carriage (vaginal swab)	**Ia**	PubMLST ID: 4489	23	**CLEAR**	**CLEAR**	NEG	NEG
**236V**	Carriage (vaginal swab)	**V**	N/A	N/A	NEG	NEG	**PLAQUE**	TURBID
**238R**	Carriage (rectal swab)	**IV**	PubMLST ID: 4490	414	NEG	NEG	NEG	NEG
**239V**	Carriage (vaginal swab)	**Ia**	N/A	N/A	TURBID	NEG	NEG	TURBID
**239R**	Carriage (rectal swab)	**Ia**	N/A	N/A	NEG	NEG	NEG	NEG
**243R**	Carriage (rectal swab)	**III**	N/A	N/A	NEG	NEG	**CLEAR**	NEG
**245V**	Carriage (vaginal swab)	**Ia**	N/A	N/A	NEG	NEG	**PLAQUE**	TURBID

**248V**	Carriage (vaginal swab)	**III**	N/A	N/A	CLEAR	CLEAR	NEG	NEG
**248R**	Carriage (rectal swab)	**III**	N/A	N/A	CLEAR	CLEAR	NEG	CLEAR
**250V**	Carriage (vaginal swab)	**II**	PubMLST ID: 4491	12	NEG	NEG	NEG	NEG
**250R**	Carriage (rectal swab)	**II**	N/A	N/A	NEG	NEG	NEG	NEG
**251V**	Carriage (vaginal swab)	**V**	N/A	N/A	NEG	NEG	PLAQUE	TURBID
**251R**	Carriage (rectal swab)	**V**	N/A	N/A	NEG	NEG	NEG	NEG
**254V**	Carriage (vaginal swab)	**VI**	PubMLST ID: 4492	1	TURBID	NEG	NEG	TURBID
**254R**	Carriage (rectal swab)	**VI**	N/A	N/A	TURBID	NEG	NEG	TURBID
**259V**	Carriage (vaginal swab)	**V**	PubMLST ID: 4493	1	NEG	NEG	NEG	TURBID
**259R**	Carriage (rectal swab)	**V**	N/A	N/A	NEG	NEG	NEG	TURBID
**263V**	Carriage (vaginal swab)	**V**	PubMLST ID: 4497	1	NEG	NEG	NEG	NEG
**263R**	Carriage (rectal swab)	**V**	PubMLST ID: 4496	1	CLEAR	NEG	NEG	NEG
**266V**	Carriage (vaginal swab)	**III**	PubMLST ID: 4498	17	NEG	NEG	NEG	TURBID
**266R**	Carriage (rectal swab)	**III**	N/A	N/A	NEG	NEG	NEG	NEG
**268V**	Carriage (vaginal swab)	**II**	PubMLST ID: 4499	1	NEG	NEG	NEG	TURBID
**272V**	Carriage (vaginal swab)	**II**	N/A	N/A	NEG	NEG	NEG	TURBID
**272R**	Carriage (rectal swab)	**II**	N/A	N/A	NEG	NEG	NEG	NEG
**273R**	Carriage (rectal swab)	**V**	PubMLST ID: 4500	1	NEG	NEG	NEG	NEG
**277V**	Carriage (vaginal swab)	**III**	PubMLST ID: 4501	19	CLEAR	CLEAR	PLAQUE	TURBID
**277R**	Carriage (rectal swab)	**III**	N/A	N/A	NEG	NEG	PLAQUE	NEG
**279V**	Carriage (vaginal swab)	**Ia**	N/A	N/A	PLAQUE	PLAQUE	NEG	CLEAR
**279R**	Carriage (rectal swab)	**Ia**	N/A	N/A	PLAQUE	PLAQUE	NEG	TURBID
**281V**	Carriage (vaginal swab)	**II**	N/A	N/A	NEG	NEG	NEG	TURBID
**281R**	Carriage (rectal swab)	**II**	N/A	N/A	NEG	NEG	NEG	TURBID
**283V**	Carriage (vaginal swab)	**Ib**	N/A	N/A	NEG	NEG	TURBID	NEG
**283R**	Carriage (rectal swab)	**V**	N/A	N/A	TURBID	NEG	PLAQUE	NEG
**284V**	Carriage (vaginal swab)	**IV**	N/A	N/A	NEG	NEG	NEG	NEG
**284R**	Carriage (rectal swab)	**IV**	N/A	N/A	NEG	NEG	NEG	NEG
**287V**	Carriage (vaginal swab)	**V**	PubMLST ID: 4502	19	NEG	NEG	NEG	NEG
**287R**	Carriage (rectal swab)	**V**	N/A	N/A	NEG	TURBID	NEG	NEG
**291V**	Carriage (vaginal swab)	**V**	PubMLST ID: 4503	41	NEG	NEG	PLAQUE	NEG
**291R**	Carriage (rectal swab)	**V**	N/A	N/A	NEG	NEG	PLAQUE	NEG
**292V**	Carriage (vaginal swab)	**III**	N/A	N/A	CLEAR	CLEAR	NEG	CLEAR

**292R**	Carriage (rectal swab)	**III**	N/A	N/A	TURBID	NEG	NEG	NEG
**297V**	Carriage (vaginal swab)	**Ia**	N/A	N/A	NEG	NEG	NEG	NEG
**297R**	Carriage (rectal swab)	**Ia**	N/A	N/A	NEG	NEG	NEG	NEG
**302V**	Carriage (vaginal swab)	**III**	N/A	N/A	CLEAR	CLEAR	CLEAR	CLEAR
**302R**	Carriage (rectal swab)	**III**	N/A	N/A	NEG	NEG	PLAQUE	TURBID
**315R**	Carriage (rectal swab)	**II**	N/A	N/A	NEG	NEG	NEG	CLEAR
**340V**	Carriage (vaginal swab)	**III**	PubMLST ID: 4505	19	NEG	NEG	CLEAR	NEG
**350R**	Carriage (rectal swab)	**III**	N/A	N/A	NEG	NEG	CLEAR	CLEAR
**371R**	Carriage (rectal swab)	**Ia**	N/A	N/A	NEG	CLEAR	PLAQUE	NEG
**373R**	Carriage (rectal swab)	**Ia**	N/A	N/A	NEG	CLEAR	PLAQUE	NEG
**376R**	Carriage (rectal swab)	**III**	N/A	N/A	TURBID	NEG	NEG	NEG
**378R**	Carriage (rectal swab)	**Ia**	N/A	N/A	TURBID + HALO	NEG	NEG	CLEAR
**399R**	Carriage (rectal swab)	**V**	N/A	N/A	TURBID + HALO	NEG	NEG	TURBID + HALO
**400V**	Carriage (vaginal swab)	**Ib**	N/A	N/A	NEG	NEG	NEG	NEG
**403V**	Carriage (vaginal swab)	**II**	N/A	N/A	TURBID	NEG	NEG	TURBID + HALO
**407V**	Carriage (vaginal swab)	**II**	N/A	N/A	NEG	NEG	NEG	NEG
**423V**	Carriage (vaginal swab)	**Ia**	N/A	N/A	CLEAR	NEG	NEG	NEG
**433V**	Carriage (vaginal swab)	**VI**	N/A	N/A	TURBID + HALO	NEG	NEG	TURBID + HALO
**434R**	Carriage (rectal swab)	**II**	N/A	N/A	CLEAR	TURBID + HALO	CLEAR	TURBID + HALO
**434V**	Carriage (vaginal swab)	**IV**	N/A	N/A	CLEAR	NEG	NEG	CLEAR
**435V**	Carriage (vaginal swab)	**II**	N/A	N/A	TURBID	TURBID	CLEAR	TURBID + HALO
**443V**	Carriage (vaginal swab)	**II**	N/A	N/A	NEG	NEG	NEG	NEG
**444V**	Carriage (vaginal swab)	**Ia**	N/A	N/A	CLEAR	NEG	CLEAR	CLEAR
**446V**	Carriage (vaginal swab)	**Ia**	N/A	N/A	CLEAR	NEG	TURBID	NEG
**454R**	Carriage (rectal swab)	**VIII**	N/A	N/A	CLEAR	NEG	PLAQUE	TURBID + HALO
**454V**	Carriage (vaginal swab)	**VIII**	N/A	N/A	TURBID	TURBID + HALO	CLEAR	NEG
**455R**	Carriage (rectal swab)	**IX**	N/A	N/A	CLEAR	NEG	NEG	NEG
**455V**	Carriage (vaginal swab)	**IX**	N/A	N/A	TURBID	NEG	NEG	NEG
**456V**	Carriage (vaginal swab)	**III**	PubMLST ID: 4512	335	TURBID	NEG	PLAQUE	CLEAR
**458R**	Carriage (rectal swab)	**Ib**	N/A	N/A	CLEAR	NEG	PLAQUE	NEG
**459V**	Carriage (vaginal swab)	**Ia**	N/A	N/A	NEG	NEG	NEG	NEG
**462V**	Carriage (vaginal swab)	**II**	PubMLST ID: 4513	28	NEG	NEG	NEG	NEG
**469V**	Carriage (vaginal swab)	**III**	PubMLST ID: 4514	1167	NEG	CLEAR	NEG	NEG

**470V**	Carriage (vaginal swab)	**Ia**	N/A	N/A	NEG	NEG	NEG	NEG
**478V**	Carriage (vaginal swab)	**II**	N/A	N/A	NEG	NEG	**CLEAR**	**CLEAR**
**480V**	Carriage (vaginal swab)	**V**	PubMLST ID: 4517	890	NEG	NEG	**CLEAR**	**CLEAR**
**481V**	Carriage (vaginal swab)	**II**	PubMLST ID: 4518	22	NEG	NEG	NEG	**CLEAR**
**482V**	Carriage (vaginal swab)	**Ib**	N/A	N/A	TURBID	NEG	**CLEAR**	**CLEAR**
**490V**	Carriage (vaginal swab)	**V**	N/A	N/A	NEG	NEG	NEG	TURBID + HALO
**493V**	Carriage (vaginal swab)	**III**	N/A	N/A	**CLEAR**	NEG	NEG	**CLEAR**
**494V**	Carriage (vaginal swab)	**II**	N/A	N/A	**CLEAR**	NEG	**PLAQUE**	TURBID + HALO
**494R**	Carriage (rectal swab)	**II**	N/A	N/A	NEG	NEG	**PLAQUE**	TURBID
**499V**	Carriage (vaginal swab)	**II**	PubMLST ID: 4521	22	**CLEAR**	NEG	NEG	**PLAQUE**
**501V**	Carriage (vaginal swab)	**III**	N/A	N/A	**CLEAR**	NEG	NEG	**CLEAR**
**506R**	Carriage (rectal swab)	**V**	N/A	N/A	NEG	NEG	NEG	NEG
**508R**	Carriage (rectal swab)	**III**	N/A	N/A	TURBID + HALO	NEG	NEG	TURBID + HALO
**509R**	Carriage (rectal swab)	**III**	N/A	N/A	**CLEAR**	NEG	NEG	NEG
**510V**	Carriage (vaginal swab)	**Ib**	N/A	N/A	**CLEAR**	NEG	TURBID	**CLEAR**
**511V**	Carriage (vaginal swab)	**Ia**	N/A	N/A	**CLEAR**	NEG	**CLEAR**	NEG
**512V**	Carriage (vaginal swab)	**V**	N/A	N/A	NEG	NEG	TURBID	NEG
**513V**	Carriage (vaginal swab)	**Ia**	N/A	N/A	**CLEAR**	TURBID + HALO	**CLEAR**	NEG
**519V**	Carriage (vaginal swab)	**V**	N/A	N/A	NEG	NEG	TURBID + HALO	TURBID + HALO
**520V**	Carriage (vaginal swab)	**V**	N/A	N/A	NEG	NEG	**CLEAR**	NEG
**528V**	Carriage (vaginal swab)	**III**	N/A	N/A	TURBID	TURBID + HALO	TURBID	TURBID + HALO
**534V**	Carriage (vaginal swab)	**III**	N/A	N/A	TURBID + HALO	NEG	NEG	TURBID + HALO
**538V**	Carriage (vaginal swab)	**Ia**	N/A	N/A	**CLEAR**	NEG	**CLEAR**	TURBID + HALO
**543R**	Carriage (rectal swab)	**V**	N/A	N/A	NEG	NEG	**CLEAR**	NEG
**544V**	Carriage (vaginal swab)	**III**	N/A	N/A	TURBID + HALO	NEG	NEG	NEG
**553R**	Carriage (rectal swab)	**III**	N/A	N/A	NEG	NEG	NEG	NEG
**553V**	Carriage (vaginal swab)	**VI**	N/A	N/A	TURBID	NEG	NEG	TURBID
**555R**	Carriage (rectal swab)	**III**	N/A	N/A	NEG	NEG	**PLAQUE**	NEG
**561V**	Carriage (vaginal swab)	**III**	N/A	N/A	TURBID + HALO	TURBID + HALO	NEG	TURBID + HALO
**562V**	Carriage (vaginal swab)	**II**	N/A	N/A	NEG	NEG	NEG	**CLEAR**
**565R**	Carriage (rectal swab)	**Ia**	N/A	N/A	NEG	NEG	NEG	NEG
**566V**	Carriage (vaginal swab)	**III**	N/A	N/A	**CLEAR**	**CLEAR**	**PLAQUE**	**PLAQUE**
**581V**	Carriage (vaginal swab)	**Ib**	N/A	N/A	**CLEAR**	NEG	**PLAQUE**	NEG

**583V**	Carriage (vaginal swab)	**Ia**	N/A	N/A	CLEAR	TURBID	CLEAR	CLEAR
**586V**	Carriage (vaginal swab)	**II**	N/A	N/A	NEG	NEG	NEG	NEG
**588V**	Carriage (vaginal swab)	**III**	N/A	N/A	TURBID	TURBID	TURBID	CLEAR
**595R**	Carriage (rectal swab)	**IV**	N/A	N/A	TURBID + HALO	NEG	NEG	TURBID + HALO
**606V**	Carriage (vaginal swab)	**III**	N/A	N/A	TURBID + HALO	NEG	TURBID + HALO	TURBID + HALO
**608R**	Carriage (rectal swab)	**II**	N/A	N/A	CLEAR	TURBID	NEG	CLEAR
**618R**	Carriage (rectal swab)	**II**	N/A	N/A	NEG	NEG	NEG	TURBID + HALO
**618V**	Carriage (vaginal swab)	**Ia**	N/A	N/A	TURBID	NEG	CLEAR	TURBID
**622V**	Carriage (vaginal swab)	**VI**	N/A	N/A	TURBID	NEG	TURBID + HALO	TURBID
**625V**	Carriage (vaginal swab)	**III**	N/A	N/A	TURBID + HALO	TURBID	NEG	TURBID
**630V**	Carriage (vaginal swab)	**VI**	N/A	N/A	TURBID	TURBID	TURBID + HALO	TURBID
**638V**	Carriage (vaginal swab)	**Ia**	N/A	N/A	NEG	TURBID	PLAQUE	NEG
**645V**	Carriage (vaginal swab)	**III**	N/A	N/A	TURBID	TURBID	TURBID	TURBID + HALO
**655V**	Carriage (vaginal swab)	**III**	N/A	N/A	TURBID + HALO	TURBID	NEG	TURBID + HALO
**656V**	Carriage (vaginal swab)	**Ia**	N/A	N/A	TURBID + HALO	TURBID + HALO	CLEAR	NEG
**657V**	Carriage (vaginal swab)	**V**	N/A	N/A	NEG	NEG	NEG	TURBID + HALO
**666R**	Carriage (rectal swab)	**II**	N/A	N/A	NEG	NEG	NEG	NEG
**666V**	Carriage (vaginal swab)	**III**	N/A	N/A	TURBID + HALO	NEG	TURBID + HALO	TURBID + HALO
**670V**	Carriage (vaginal swab)	**Ia**	N/A	N/A	TURBID + HALO	TURBID + HALO	PLAQUE	TURBID
**671R**	Carriage (rectal swab)	**Ib**	N/A	N/A	NEG	NEG	PLAQUE	NEG
**672V**	Carriage (vaginal swab)	**Ia**	N/A	N/A	NEG	NEG	NEG	TURBID
**675V**	Carriage (vaginal swab)	**II**	N/A	N/A	TURBID	NEG	CLEAR	TURBID
**679R**	Carriage (rectal swab)	**II**	N/A	N/A	TURBID	TURBID	TURBID	TURBID + HALO
**680R**	Carriage (rectal swab)	**Ia**	N/A	N/A	NEG	NEG	NEG	NEG
**685V**	Carriage (vaginal swab)	**III**	N/A	N/A	NEG	NEG	NEG	NEG
**690V**	Carriage (vaginal swab)	**Ia**	N/A	N/A	TURBID	TURBID + HALO	NEG	TURBID
**691V**	Carriage (vaginal swab)	**V**	N/A	N/A	TURBID + HALO	TURBID	CLEAR	TURBID + HALO
**692V**	Carriage (vaginal swab)	**Ia**	N/A	N/A	TURBID	NEG	NEG	TURBID
**695V**	Carriage (vaginal swab)	**Ia**	N/A	N/A	NEG	NEG	NEG	NEG
**698R**	Carriage (rectal swab)	**Ia**	N/A	N/A	NEG	NEG	NEG	NEG
**704R**	Carriage (rectal swab)	**Ia**	N/A	N/A	CLEAR	NEG	NEG	TURBID + HALO
**725V**	Carriage (vaginal swab)	**Ia**	N/A	N/A	TURBID + HALO	TURBID + HALO	NEG	NEG
**738V**	Carriage (vaginal swab)	**Ib**	N/A	N/A	NEG	TURBID + HALO	PLAQUE	NEG

**746V**	Carriage (vaginal swab)	**Ia**	N/A	N/A	TURBID + HALO	TURBID	NEG	NEG
**748V**	Carriage (vaginal swab)	**Ia**	N/A	N/A	**CLEAR**	NEG	NEG	TURBID + HALO
**750R**	Carriage (rectal swab)	**V**	N/A	N/A	TURBID	NEG	NEG	TURBID + HALO
**754R**	Carriage (rectal swab)	**Ia**	N/A	N/A	NEG	NEG	NEG	NEG
**757V**	Carriage (vaginal swab)	**Ia**	N/A	N/A	TURBID + HALO	TURBID + HALO	NEG	TURBID + HALO
**765V**	Carriage (vaginal swab)	**V**	N/A	N/A	TURBID	NEG	NEG	TURBID
**771V**	Carriage (vaginal swab)	**V**	N/A	N/A	TURBID	NEG	**PLAQUE**	TURBID
**774R**	Carriage (rectal swab)	**II**	N/A	N/A	TURBID	NEG	NEG	**CLEAR**
**774V**	Carriage (vaginal swab)	**Ib**	N/A	N/A	TURBID	NEG	**PLAQUE**	TURBID
**777V**	Carriage (vaginal swab)	**Ia**	N/A	N/A	TURBID	TURBID	NEG	NEG
**782V**	Carriage (vaginal swab)	**II**	N/A	N/A	NEG	NEG	NEG	TURBID + HALO
**784V**	Carriage (vaginal swab)	**IV**	N/A	N/A	TURBID	**CLEAR**	TURBID	**CLEAR**
**786V**	Carriage (vaginal swab)	**Ia**	N/A	N/A	TURBID	NEG	**CLEAR**	TURBID + HALO
**787V**	Carriage (vaginal swab)	**Ia**	N/A	N/A	**CLEAR**	**CLEAR**	NEG	NEG
**798V**	Carriage (vaginal swab)	**Ia**	N/A	N/A	NEG	TURBID	**PLAQUE**	TURBID
**801V**	Carriage (vaginal swab)	**II**	N/A	N/A	TURBID	TURBID	TURBID	TURBID
**805R**	Carriage (rectal swab)	**Ib**	N/A	N/A	NEG	NEG	**PLAQUE**	NEG
**808R**	Carriage (rectal swab)	**Ib**	N/A	N/A	NEG	NEG	NEG	NEG
**808V**	Carriage (vaginal swab)	**VI**	N/A	N/A	NEG	TURBID	NEG	TURBID
**809V**	Carriage (vaginal swab)	**Ib**	N/A	N/A	TURBID + HALO	NEG	NEG	**CLEAR**
**1_NEO**	Invasive (blood culture)	**IV**	PubMLST ID: 4633	196	NEG	**CLEAR**	NEG	**CLEAR**
**2_NEO**	Invasive (blood culture)	**V**	PubMLST ID: 4635	1	**CLEAR**	**CLEAR**	NEG	**CLEAR**
***3_NEO**	Invasive (blood culture)	**III**	PubMLST ID: 4636	19	**CLEAR**	**CLEAR**	NEG	**CLEAR**
**4_NEO**	Invasive (blood culture)	**Ia**	PubMLST ID: 4637	509	**CLEAR**	**CLEAR**	NEG	**CLEAR**
***5_NEO**	Invasive (blood culture)	**Ib**	PubMLST ID: 4638	1	TURBID	**CLEAR**	NEG	TURBID
**6_NEO**	Invasive (blood culture)	**Ib**	PubMLST ID: 4639	8	**CLEAR**	**CLEAR**	NEG	**CLEAR**
**7_NEO**	Invasive (blood culture)	**III**	PubMLST ID: 4640	17	**CLEAR**	**CLEAR**	NEG	**CLEAR**
***8_NEO**	Invasive (blood culture)	**Ia**	PubMLST ID: 4641	23	**CLEAR**	**CLEAR**	NEG	**CLEAR**
***9_NEO**	Invasive (blood culture)	**V**	PubMLST ID: 4642	585	**CLEAR**	**CLEAR**	NEG	TURBID
**10_NEO**	Invasive (blood culture)	**VI**	PubMLST ID: 4634	1	TURBID	**CLEAR**	NEG	TURBID
***Kong_4**	Reference (NCTC 12907)	**Ia**	PubMLST ID: 4629	7	NEG	NEG	NEG	NEG
***Kong_7**	Reference (ATCC 12400)	**Ia**	PubMLST ID: 4630	25	**PLAQUE**	**PLAQUE**	NEG	NEG
***Kong_8**	Reference (ATCC 12401)	**Ib**	PubMLST ID: 4631	6	NEG	NEG	NEG	NEG

***Kong_10**	Reference (NZRM 910)	**Ia**	PubMLST ID: 4613	7	NEG	NEG	**PLAQUE**	NEG
***Kong_11**	Reference (NCTC 11079)	**II**	PubMLST ID: 4614	19	NEG	NEG	NEG	NEG
***Kong_16**	Reference (BM-110)	**III**	N/A	N/A	NEG	NEG	NEG	NEG
***Kong_18**	Reference (PubMLST ID: 4619)	**IV**	PubMLST ID: 4619	2	NEG	NEG	NEG	NEG
***Kong_20**	Reference (ATCC 49446)	**IV**	PubMLST ID: 4620	67	NEG	NEG	NEG	NEG
***Kong_22**	Reference (NCTC 9828)	**NT**	PubMLST ID: 4622	1	NEG	NEG	NEG	NEG
***Kong_23**	Reference (ATCC BAA-23)	**V**	PubMLST ID: 4623	26	NEG	NEG	NEG	NEG
***Kong_24**	Reference (ATCC 49447)	**V**	PubMLST ID: 4624	14	NEG	NEG	NEG	NEG
***Kong_25**	Reference (PubMLST ID: 4625)	**VI**	PubMLST ID: 4625	67	NEG	NEG	NEG	NEG
***Kong_26**	Reference (NZRM 2834)	**VI**	PubMLST ID: 4626	1	NEG	NEG	NEG	NEG
***Kong_27**	Reference (PubMLST ID: 4627)	**VII**	PubMLST ID: 4627	1	NEG	NEG	NEG	NEG
***Kong_28**	Reference (PubMLST ID: 4628)	**VIII**	PubMLST ID: 4628	1	NEG	NEG	NEG	**PLAQUE**
**Specificity testing**								
S. pyogenes	ATCC 12203				NEG	NEG	NEG	NEG
S. equinus	ATCC 15351				NEG	NEG	NEG	NEG
S. mitis	M2611				NEG	NEG	NEG	NEG
S. dysagalactiae subsp. equisimilis	NCTC 5371				NEG	NEG	NEG	NEG
S. dysgalactiae	Clinical (rectal swab)				NEG	NEG	NEG	NEG
S. gordonii	ATCC 10558				NEG	NEG	NEG	NEG
S. anginosus	NCTC 8037				NEG	NEG	NEG	NEG
S. salivarius	Clinical (vaginal swab)				**CLEAR**	**CLEAR**	NEG	**CLEAR**
S. aureus	ATCC 9144				NEG	NEG	NEG	NEG
S. saprophyticus	ATCC 15305				NEG	NEG	NEG	NEG
S. epidermidis	ATCC 14990				NEG	NEG	NEG	NEG
E. faecalis	ATCC 19433				NEG	NEG	NEG	NEG
L. crispatus	BEI HM-637				NEG	NEG	NEG	NEG
L. gasseri	BEI HM-104				NEG	NEG	NEG	NEG
L. jensenii	BEI HM-105				NEG	NEG	NEG	NEG
L. johnsonii	BEI HM-643				NEG	NEG	NEG	NEG
L. rhamnosus	BEI HM-106				NEG	NEG	NEG	NEG

N/A, Not available; PLAQUE, Plaque formation; CLEAR, Clearing zones; TURBID, Turbid zones; HALO; Clear border observed as a halo.
