# Supplementary material for: Host range, morphological and genomic characterisation of bacteriophages with activity against clinical Streptococcus agalactiae isolates
Source: PLoS One. 2020 Jun 23;15(6):e0235002. doi: 10.1371/journal.pone.0235002 (PMC7310703; doi:10.1371/journal.pone.0235002)
Supplement: S2 Table — (PDF) [file pone.0235002.s004.pdf]

S2 Table. Genome annotations of *S. agalactiae* phages LF1- LF4.

ORF	Min	Max	Length (bp)	Direction	Transferred from	Transferred similarity	Product	
							Geneious (BLASTN)	RASTtk
LF1 Phage								
ORF1	269	1138	870	Forward	CP000114	99.77%	Conserved hypothetical protein	Phage protein
ORF2	1158	1439	282	Forward	CP000114	99.29%	Conserved hypothetical protein	
	1182	1439	258	Forward				Phage protein
ORF3	1518	1637	120	Forward				Phage protein
ORF4	1798	2037	240	Forward	LT714196	99.58%	Hypothetical protein	Phage protein (ACLAME 173)
ORF5	2068	2232	165	Forward	LT714196	100.00%	Hypothetical protein	Hypothetical protein
ORF6	2229	2498	270	Forward	LT714196, LT714196	99.26% – 92.22%	Hypothetical protein	Phage protein
ORF7	2502	2645	144	Forward	LT714196	100.00%	Hypothetical protein	Hypothetical protein
ORF8	2642	3154	513	Forward	LT714196, LT714196	99.81% – 84.21%	Hypothetical protein	Phage protein
ORF9	3175	3474	300	Forward	LT714196	99.67%	Hypothetical protein	Phage protein
ORF10	3492	3665	174	Forward	LT714196	100.00%	Hypothetical protein	Hypothetical protein
ORF11	3662	3856	195	Forward	LT714196	98.46%	Hypothetical protein	Hypothetical protein
ORF12	3853	4119	267	Forward	LT714196, LT714196, AP012335	98.50% – 94.76%	Hypothetical protein; phage protein	Hypothetical protein
ORF13	4507	4935	429	Forward	CP000114	99.77%	Conserved hypothetical protein	
	4510	4935	426	Forward				Phage protein
ORF14	5241	5468	228	Reverse				Hypothetical protein
ORF15	5507	5881	375	Forward	CP000114	100.00%	Prophage LambdaSa03, HNH endonuclease family protein	Phage-associated HNH homing endonuclease
ORF16	6032	6388	357	Forward	AP012335, NC_004586	87.68%	Hypothetical protein; phage protein	FIG018599: Phage-associated protein
ORF17	6385	7653	1269	Forward	AP012335, NC_004586	81.40% – 81.32%	Putative structural protein - phage associated; putative structural protein	Phage portal protein; Phage capsid and scaffold
ORF18	7646	8866	1221	Forward	CP000114	99.92%	Conserved hypothetical protein	
	7646	8863	1218					Phage protein
ORF19	8866	9054	189	Forward	CP000114	99.47%	Hypothetical protein	Hypothetical protein

ORF20	9162	10577	1416	Forward	AP012335, NC_004586	85.73%	Phage terminase; putative terminase	Phage terminase
ORF21	10658	11122	465	Forward	CP000114	100.00%	Conserved hypothetical protein	Phage capsid and scaffold
ORF22	11125	12027	903	Forward	CP000114	99.78%	Prophage LambdaSa03, structural protein, putative	Phage major capsid protein
ORF23	12024	12239	216	Forward	CP000114	100.00%	Conserved hypothetical protein	FIG021300: Phage-associated protein
ORF24	12253	12684	432	Forward	LT714196	81.48%	Phage protein Gp19/Gp15/Gp42	FIG017897: Phage protein
ORF25	12635	12973	339	Forward	CP000114	100.00%	Conserved hypothetical protein	FIG018632: Phage-associated protein
ORF26	12933	13202	270	Forward	NC_004586	98.52%	Hypothetical protein	
	12966	13202	237	Forward				FIG020717: Phage-associated protein
ORF27	13203	13538	336	Forward	AP012335, NC_004586	98.81%	Hypothetical protein; phage protein	FIG018217: Phage-associated protein
ORF28	13548	14105	558	Forward	LT714196	83.33%	Prophage LambdaSa1, structural protein	Phage major tail protein
ORF29	14105	14350	246	Forward	CP000114	100.00%	Conserved hypothetical protein	Phage protein
ORF30	14365	14736	372	Forward	CP000114	100.00%	Conserved hypothetical protein	Phage protein
ORF31	14736	16748	2013	Forward	CP000114	99.85%	Prophage LambdaSa03, pblA protein, internal deletion	Phage minor tail protein
ORF32	16742	18274	1533	Forward	LT714196	84.87%	Prophage LambdaSa03, tail component	Putative minor structural protein
ORF33	18275	22396	4122	Forward	CP000114	99.98%	Prophage LambdaSa03, minor structural protein, putative	Phage tail fibers
ORF34	22397	24409	2013	Forward	LT714196	97.47%	Prophage LambdaSa03, minor structural protein	Structural protein
ORF35	24421	24813	393	Forward	LT714196	93.89%	Hypothetical protein	Hypothetical protein
ORF36	24836	24964	129	Forward	CP000114	100.00%	Conserved hypothetical protein	
	24764	24964	201	Forward				Hypothetical protein
ORF37	24973	25275	303	Forward	LT714196	99.34%	Phage protein	Phage protein (ACLAME 358)
ORF38	25268	25495	228	Forward	LT714196, NC_004589, DQ864624, NC_004584, NC_009819,	92.98% – 88.16%	prophage LambdaSa03, holin; putative holin; holin	Phage holin

					NC_004585, AP012335			
ORF39	25504	25620	117	Forward	JX409894, LT714196, JX409895	99.15% – 94.02%	Hypothetical protein	Hypothetical protein
ORF40	25621	26952	1332	Forward	LT714196, JX409895, JX409894	98.65% – 93.84%	Phage lysin, N-acetylmuramoyl-L-alanine amidase; putative lysin	Phage lysin, N-acetylmuramoyl-L-alanine amidase
ORF41	27214	27384	171	Reverse	CP021869, LT714196	100.00%	Hypothetical protein	FIG01116037: hypothetical protein
ORF42	27482	27766	285	Forward				Hypothetical protein
ORF43	27803	27982	180	Forward	CP021869, LT714196	100.00%	Paratox	Paratox
ORF44	28279	29421	1143	Reverse	CP021869, LT714196	96.33%	Prophage LambdaSa03, site-specific recombinase phage integrase; site-specific integrase	Phage integrase
ORF45	29549	29758	210	Reverse	CP000114	100.00%	Conserved hypothetical protein	
ORF46	29810	30190	381	Reverse	JX409894, LT714196	99.74% – 95.80%	Putative CI-like repressor; hypothetical protein	Phage protein
ORF47	30177	30536	360	Reverse	CP000114	99.44%	Prophage LambdaSa03, transcriptional regulator, Cro/CI family	Phage cI repressor (ACLAME 5)
ORF48	30728	30946	219	Forward	KT388093, KY349816, NC_004588, NC_028697	80.82%	Cro-like transcriptional regulator; cro family anti-repressor; putative Cro-like repressor	Transcriptional regulator
ORF49	31044	31376	333	Forward	JX409895, LF4 Phage - Complete	99.70%	Hypothetical protein	Phage protein
ORF50	31481	31816	336	Reverse	LT714196	98.21%	Hypothetical protein	Hypothetical protein
ORF51	31962	32147	186	Forward	JX409895, JX409894	98.39% – 95.70%	Putative DNA-binding phage protein	Putative DNA-binding phage protein
ORF52	32226	32537	312	Forward	CP021869, LT714196	94.23%	Excisionase; putative excisionase	Phage excisionase
ORF53	32542	32691	150	Forward	LT714196, LT714196	99.33% – 83.33%	Hypothetical protein	
ORF54	32685	32774	90	Forward				Hypothetical protein
ORF55	32767	32967	201	Forward	JX409895	100.00%	Hypothetical protein	Hypothetical protein

ORF56	32964	33254	291	Forward	CP000114	100.00%	Conserved hypothetical protein	Hypothetical phage protein
ORF57	33241	33924	684	Forward	CP000114	99.85%	Conserved hypothetical protein TIGR01618	ORF46
ORF58	33999	35363	1365	Forward	CP000114	100.00%	Prophage LambdaSa03, helicase, putative	Putative helicase
ORF59	35368	35850	483	Forward	CP000114	100.00%	Conserved hypothetical protein	Phage protein
ORF60	35868	37421	1554	Forward	CP000114	99.74%	Conserved hypothetical protein/bacteriophage resistance protein	Phage protein
LF2 Phage								
ORF1	84	551	468	Forward				Phage terminase, small subunit
ORF2	566	2320	1755	Forward	LT714196	99.89%	Phage terminase large subunit	Phage terminase, large subunit
ORF3	2320	2487	168	Forward	LT714196	100.00%	Hypothetical protein	Phage protein
ORF4	2480	2683	204	Forward	LT714196	100.00%	Phage protein	Phage protein
ORF5	2714	3934	1221	Forward	LT714196	100.00%	Phage portal protein	Phage portal protein
ORF6	3912	4577	666	Forward	LT714196	99.85%	ATP-dependent Clp protease protease subunit	Prophage Clp protease-like protein
ORF7	4601	5785	1185	Forward	LT714196	99.92%	Phage capsid family protein	Phage capsid protein
ORF8	5799	5960	162	Forward	JX409894, JX409895	100.00%	Hypothetical protein	Putative capsid protein (ACLAME 311)
ORF9	5963	6265	303	Forward	JX409894, JX409895, LT714196	100.00%	Putative head-tail connector protein; unknown phage protein	Phage DNA packaging protein (ACLAME 138)
ORF10	6262	6609	348	Forward	LT714196	100.00%	Phage head-tail joining protein	Phage protein
ORF11	6606	6971	366	Forward				Phage capsid and scaffold (ACLAME 58)
ORF12	6968	7405	438	Forward				Phage protein
ORF13	7421	8044	624	Forward	LT714196	100.00%	Phage major tail protein	Phage major tail protein
ORF14	8098	8418	321	Forward	LT714196	100.00%	Hypothetical protein	Phage protein
ORF15	8502	8615	114	Forward				Phage protein
ORF16	8628	12569	3942	Forward	LT714196, JX409894, JX409895	99.95% – 90.36%	Chromosome partition protein Smc; putative phage tape measure protein; putative tail tape measure protein	Phage tail length tape-measure protein
ORF17	12566	13897	1332	Forward				Putative minor structural protein
ORF18	13954	14076	123	Forward				Putative minor structural protein
ORF19	14067	17993	3927	Forward				Phage tail fibers

ORF20	18207	20198	1992	Forward	LT714196	100.00%	Prophage LambdaSa03, minor structural protein	Structural protein
ORF21	20212	20538	327	Forward	JX409894, LT714196, JX409895	99.69% – 99.08%	Hypothetical protein	Hypothetical protein
ORF22	20567	20725	159	Forward				Hypothetical protein
ORF23	20744	21031	288	Forward	LT714196	100.00%	Phage protein	Phage protein
ORF24	21033	21287	255	Forward	JX409894, JX409895, LT714196	100.00%	Holin; putative holin	Phage holin
ORF25	21296	21412	117	Forward	LT714196	100.00%	Hypothetical protein	Hypothetical protein
ORF26	21413	22756	1344	Forward	LT714196	99.93%	Phage lysin, N-acetylmuramoyl-L-alanine amidase	Phage lysin, N-acetylmuramoyl-L-alanine amidase
ORF27	23103	23300	198	Forward	LT714196	100.00%	CsbD-like superfamily protein	Hypothetical protein
ORF28	23412	23594	183	Forward	LT714196	100.00%	Paratox	Paratox
ORF29	23864	24961	1098	Reverse	LT714196, AP012335	99.82% – 95.26%	Mobile element protein; DNA integration/recombination/inversion protein	Integrase
ORF30	25134	25748	615	Reverse	LT714196	100.00%	Hypothetical protein	Hypothetical protein
ORF31	25880	26659	780	Reverse	LT714196	99.74%	Putative repressor-phage associated	Predicted transcriptional regulator
ORF32	27158	27370	213	Reverse	JX409895, LT714196	100.00%	Hypothetical protein	Hypothetical protein
ORF33	27429	27587	159	Forward	LT714196	100.00%	FIG01115240: hypothetical protein	FIG01115240: hypothetical protein
ORF34	27685	28326	642	Reverse	JX409895, LT714196	100.00%	Hypothetical protein	Hypothetical protein
ORF35	28543	28878	336	Reverse	LT714196	99.70%	Hypothetical protein	Hypothetical protein
ORF36	28954	29160	207	Forward	LT714196	100.00%	DNA-binding protein	Transcriptional regulator, Cro/CI family
ORF37	29214	30173	960	Forward	LT714196	100.00%	Hypothetical protein	Hypothetical protein
ORF38	30166	30687	522	Forward	LT714196	100.00%	Hypothetical protein	Phage antirepressor protein
ORF39	30706	30897	192	Forward	LT714196	100.00%	Hypothetical protein	Hypothetical protein
ORF40	30925	31101	177	Reverse	LT714196	100.00%	Hypothetical protein	Hypothetical protein
ORF41	31195	31392	198	Forward				Conserved hypothetical protein - phage associated

ORF42	31471	31728	258	Forward	LT714196	100.00%	FIG01116860: hypothetical protein	Phage protein
ORF43	31758	31889	132	Forward	LT714196	100.00%	Hypothetical protein	Phage protein
ORF44	32026	32196	171	Forward	LT714196	99.42%	Prophage Sa05, CopG family transcriptional regulator	Hypothetical protein
ORF45	32377	32649	273	Forward	LT714196	99.63%	Hypothetical protein	Hypothetical protein
ORF46	32639	33019	381	Reverse	LT714196	100.00%	Hypothetical protein	Hypothetical protein
ORF47	33075	33518	444	Forward	LT714196	100.00%	Hypothetical protein	Phage protein
ORF48	33511	33633	123	Forward				Hypothetical protein
ORF49	33634	34536	903	Forward	LT714196	100.00%	DNA replication protein dnaD	DNA replication protein DnaC
ORF50	34546	35388	843	Forward	LT714196	100.00%	Prophage LambdaSa2, DNA replication protein DnaC	DNA replication protein DnaC
ORF51	35388	35534	147	Forward	LT714196	100.00%	Hypothetical protein	Phage protein
ORF52	35524	35799	276	Forward	LT714196	100.00%	Hypothetical protein	Hypothetical protein
ORF53	35786	36040	255	Forward	LT714196	100.00%	FIG01116817: hypothetical protein	Phage protein
ORF54	36043	36204	162	Forward	LT714196	100.00%	Hypothetical protein	Hypothetical protein
ORF55	36206	36535	330	Forward	LT714196	100.00%	Hypothetical protein	Phage protein
ORF56	36538	37500	963	Forward	LT714196, JX409894	99.79% – 94.08%	Putative recombinase-phage associated; putative RecT protein	Recombinational DNA repair protein RecT (prophage associated)
ORF57	37497	38294	798	Forward	LT714196	100.00%	Hypothetical protein phage associated	Phage protein
ORF58	38456	38653	198	Forward	LT714196	100.00%	Hypothetical protein	Hypothetical protein
ORF59	38643	39047	405	Forward				Phage Holliday junction resolvase
	38643	39119	477	Forward	LT714196	99.58%	Phage Holliday junction resolvase	
ORF60	39304	39456	153	Forward				Hypothetical protein
ORF61	39468	39764	297	Forward	LT714196	100.00%	Hypothetical protein	Phage protein
ORF62	39748	39987	240	Forward	LT714196	100.00%	Hypothetical protein	Phage protein (ACLAME 173)
ORF63	40018	40182	165	Forward	LT714196	100.00%	Hypothetical protein	Hypothetical protein
ORF64	40179	40448	270	Forward	LT714196	100.00%	Hypothetical protein	Phage protein
ORF65	40452	40595	144	Forward	LT714196	100.00%	Hypothetical protein	Hypothetical protein
ORF66	40592	41104	513	Forward	LT714196	100.00%	Hypothetical protein	Phage protein
ORF67	41125	41424	300	Forward	LT714196	99.67%	Hypothetical protein	Hypothetical protein
ORF68	41442	41615	174	Forward	LT714196	100.00%	Hypothetical protein	Hypothetical protein
ORF69	41612	41806	195	Forward	LT714196	100.00%	Hypothetical protein	Hypothetical protein
ORF70	41803	42069	267	Forward	LT714196	100.00%	Hypothetical protein	Hypothetical protein

ORF71	42458	42892	435	Forward	LT714196	100.00%	FIG01114733: hypothetical protein	Phage protein
RNA1	43058	43136	79	Forward				tRNA-Tyr-ATA
ORF72	43448	43576	129	Forward	LT714196	100.00%	Hypothetical protein	Hypothetical protein
ORF73	43630	44007	378	Reverse	LT714196	100.00%	Phage protein	Phage protein
ORF74	44060	44245	186	Reverse	LT714196	100.00%	YcfA-like protein	Phage protein
ORF75	44346	44681	336	Forward	LT714196	100.00%	HNH endonuclease	Phage endonuclease
ORF76	44687	44767	81	Reverse				Hypothetical protein (partial)
LF3 Phage								
ORF1	57	536	480	Forward				FIG01116616: hypothetical protein
ORF2	529	2241	1713	Forward	CP021869, KY363359, KY065484, KM882824, NC_027396, KY065449, KY065482, KT337370, KY065481, KY065483, KY065489, NC_030946	92.35% – 88.32%	Terminase; terminase large subunit	Phage terminase, large subunit
ORF3	2250	3386	1137	Forward	KY363359, CP021869	96.24% – 96.15%	Portal protein; phage portal protein	FIG01118414: hypothetical protein
ORF4	3438	3980	543	Forward	KY363359, CP021869, KY065484, KY065449, KM882824, KT337370, KY065489, NC_027396, NC_030946, KY065481, KY065482, KY065483	97.97% – 87.29%	Prohead protease; peptidase U35; protease; prohead maturation protease	Prophage LambdaSa2, protease, putative

ORF5	3991	5253	1263	Forward	CP021869, KY363359	96.99% – 96.83%	Phage major capsid protein; major capsid protein	
	3991	5250	1260	Forward				Conserved domain protein
ORF6	5273	5608	336	Forward	CP021869, KY363359	98.21% – 97.92%	Hypothetical protein	Glycerate kinase (EC 2.7.1.31)
ORF7	5605	5910	306	Forward	CP021869, KY363359, KM882824, NC_027396, KY065449, KY065481, KY065482, KY065483, KY065484, KY065489, KT337370, NC_030946	98.37% – 79.41%	Phage head-tail adapter protein; head-tail adapter protein; head-tail adaptor; hypothetical protein	FIG01114606: hypothetical protein
ORF8	5910	6257	348	Forward	KY363359, CP021869, KM882824, KY065484, NC_027396, KT337370, KY065449, KY065481, KY065482, KY065483, KY065489, NC_030946	96.84% – 82.47%	Hypothetical protein	
ORF9	6244	6588	345	Forward	CP021869, KY363359	96.52% – 95.07%	Hypothetical protein; putative tail-component	FIG01116328: hypothetical protein
ORF10	6603	7286	684	Forward	CP021869, KY363359	97.95%	Phage tail protein; tail protein	FIG01118296: hypothetical protein
ORF11	7286	7759	474	Forward	KY363359, CP021869, KY065484, KM882824, NC_027396,	87.84% – 86.58%	Hypothetical protein	FIG01114006: hypothetical protein

					KT337370, KY065449, KY065481, KY065482, KY065483, NC_030946			
ORF12	7801	7929	129	Forward	CP021869, KM882824, NC_027396	94.57% – 78.29%	Hypothetical protein	FIG01115934: hypothetical protein
ORF13	7948	10669	2722	Forward	CP021869, KY363359	79.69% – 79.63%	Phage tail tape measure protein; tail tape-measure protein	
	7948	10497	2550	Forward				Phage tail length tape-measure protein
ORF14	10494	11216	723	Forward	CP021869, KY363359	96.40% – 95.57%	Phage tail protein; tail protein	FIG01115816: hypothetical protein
ORF15	11217	14891	3675	Forward	CP021869, KY363359	97.61% – 97.03%	Hypothetical protein; tail endopeptidase	Phage hyaluronidase
ORF16	14908	15138	231	Forward	KY363359, CP021869	99.57% – 98.70%	Hypothetical protein	Conserved domain protein
ORF17	15141	15479	339	Forward	CP021869, KY363359	93.22%	Hypothetical protein	Phage protein
ORF18	15513	15923	411	Forward	CP021869, KY363359	96.59%	Hypothetical protein	Hypothetical protein
ORF19	15927	16256	330	Forward	CP021869, KY363359	93.03% – 92.73%	Phage holin; holin	Phage holin
ORF20	16260	17699	1440	Forward	KT388093, KY349816, NC_028697	78.35%	N-acetylmuramoyl-L-alanine amidase; Lysin	Phage lysin, N-acetylmuramoyl-L-alanine amidase (EC 3.5.1.28)
ORF21	17907	18092	186	Forward	KY363359	100.00%	Toxin	Hypothetical protein
ORF22	18153	18557	405	Forward	KY363359, CP021869, KC348604	98.52% – 88.89%	Antitoxin; HicB family protein; toxin-antitoxin system, antitoxin component, HicB family	Phage-related protein
ORF23	18842	19909	1068	Reverse	CP021869	100.00%	Site-specific integrase	Integrase
ORF24	20033	20437	405	Reverse	CP021869	100.00%	Hypothetical protein	Phage protein
ORF25	20459	21205	747	Reverse	CP021869	100.00%	Hypothetical protein	Hypothetical protein
ORF26	21214	21594	381	Reverse	CP021869	100.00%	Transcriptional regulator	Transcriptional regulator
ORF27	22029	22499	471	Reverse	CP021869	100.00%	Hypothetical protein	Hypothetical protein

ORF28	22560	22772	213	Forward	KY363359	100.00%	Transcriptional regulator	Phage protein (ACLAME 752)
ORF29	22772	23113	342	Forward	KY363359	100.00%	Hypothetical protein	Hypothetical protein
ORF30	23160	23690	531	Forward	KY363359	100.00%	HNH homing endonuclease	HNH homing endonuclease
ORF31	23690	23974	285	Forward	KY363359	100.00%	Hypothetical protein	Phage protein
ORF32	23998	24216	219	Forward	KY363359	100.00%	Hypothetical protein	Hypothetical protein
ORF33	24225	25556	1332	Forward	CP021869	100.00%	Replicative DNA helicase	Replicative DNA helicase (DnaB) (EC 3.6.4.12)
ORF34	25558	26160	603	Forward	KY363359	100.00%	Hypothetical protein	Phage protein
ORF35	26138	26884	747	Forward	KY363359	100.00%	DNA replication protein	Phage replication initiation protein
ORF36	26885	27706	822	Forward	KY363359, CP021869	99.88% – 95.13%	DNA replication protein	Phage DNA helicase loader (ACLAME 19)
ORF37	27706	27954	249	Forward	KY363359	100.00%	Hypothetical protein	Hypothetical protein
ORF38	27947	28441	495	Forward	KY363359	100.00%	Nucleoside triphosphate pyrophosphohydrolase	Phage DNA binding protein
ORF39	28438	28767	330	Forward	KY363359	100.00%	Hypothetical protein	Phage protein (ACLAME 481)
ORF40	28767	29186	420	Forward	KY363359	100.00%	Hypothetical protein	Phage protein
ORF41	29183	29389	207	Forward	KY363359	100.00%	Hypothetical protein	Hypothetical protein
ORF42	29379	29795	417	Forward	KY363359	100.00%	Single-stranded DNA-binding protein	Single-stranded DNA-binding protein
ORF43	29805	30065	261	Forward	KY363359	100.00%	Hypothetical protein	Phage protein
ORF44	30062	30409	348	Forward	KY363359	100.00%	Cro/CI family transcriptional regulator	Prophage LambdaSa2, transcriptional regulator, Cro/CI family
ORF45	30406	30870	465	Forward	KY363359	100.00%	Hypothetical protein	Hypothetical protein
ORF46	30980	31522	543	Forward	KY363359	100.00%	Site-specific recombinase	Prophage LambdaSa2, site-specific recombinase, phage integrase family
ORF47	32020	32205	186	Reverse				Hypothetical protein
LF4 Phage								
ORF1	269	1138	870	Forward	CP000114	100.00%	Conserved hypothetical protein	Phage protein
ORF2	1158	1439	282	Forward	CP000114	100.00%	Conserved hypothetical protein	Phage protein
ORF3	1518	1814	297	Forward	LT714196	100.00%	Hypothetical protein	Phage protein
ORF4	1798	2037	240	Forward	LT714196	100.00%	Hypothetical protein	Phage protein (ACLAME 173)
ORF5	2068	2232	165	Forward	LT714196	100.00%	Hypothetical protein	Hypothetical protein

ORF6	2229	2498	270	Forward	LT714196	100.00%	Hypothetical protein	Phage protein
ORF7	2502	2645	144	Forward	LT714196	100.00%	Hypothetical protein	Hypothetical protein
ORF8	2642	3154	513	Forward	LT714196	100.00%	Hypothetical protein	Phage protein
ORF9	3175	3474	300	Forward	LT714196	100.00%	Hypothetical protein	Hypothetical protein
ORF10	3492	3665	174	Forward	LT714196	100.00%	Hypothetical protein	Hypothetical protein
ORF11	3662	3856	195	Forward	LT714196	98.46%	Hypothetical protein	Hypothetical protein
ORF12	3853	4119	267	Forward	LT714196, LT714196, AP012335	98.50% – 94.76%	Hypothetical protein; phage protein	Hypothetical protein
ORF13	4507	4935	429	Forward	CP000114	100.00%	Conserved hypothetical protein	
	4510	4935	426	Forward				Phage protein
ORF14	5241	5468	228	Reverse				Hypothetical protein
ORF15	5507	5881	375	Forward	CP000114	100.00%	Prophage LambdaSa03, HNH endonuclease family protein	Phage-associated HNH homing endonuclease
ORF16	6032	6388	357	Forward	AP012335, NC_004586	87.96%	Hypothetical protein; phage protein	FIG018599: Phage-associated protein
ORF17	6385	7653	1269	Forward	AP012335, NC_004586	81.40% – 81.32%	Putative structural protein - phage associated; putative structural protein	Phage portal protein; Phage capsid and scaffold
ORF18	7646	8866	1221	Forward	CP000114	100.00%	Conserved hypothetical protein	Phage protein
ORF19	8866	9054	189	Forward	CP000114	100.00%	Hypothetical protein	Hypothetical protein
ORF20	9162	10577	1416	Forward	AP012335, NC_004586	85.81%	Phage terminase; putative terminase	Phage terminase
ORF21	10658	11122	465	Forward	CP000114	100.00%	Conserved hypothetical protein	Phage capsid and scaffold
ORF22	11125	12027	903	Forward	CP000114	100.00%	Prophage LambdaSa03, structural protein, putative	Phage major capsid protein
ORF23	12024	12239	216	Forward	CP000114	100.00%	Conserved hypothetical protein	FIG021300: Phage-associated protein
ORF24	12253	12684	432	Forward	LT714196	81.71%	Phage protein Gp19/Gp15/Gp42	FIG017897: Phage protein
ORF25	12635	12973	339	Forward	CP000114	100.00%	Conserved hypothetical protein	FIG018632: Phage-associated protein
ORF26	12966	13202	237	Forward	AP012335	98.31%	Phage protein	FIG020717: Phage-associated protein
ORF27	13203	13538	336	Forward	AP012335, NC_004586	98.81%	Hypothetical protein; phage protein	FIG018217: Phage-associated protein
ORF28	13548	14105	558	Forward	LT714196	83.33%	Prophage LambdaSa1, structural protein	Phage major tail protein

ORF29	14105	14350	246	Forward	CP000114	100.00%	Conserved hypothetical protein	Phage protein
ORF30	14365	14736	372	Forward	CP000114	100.00%	Conserved hypothetical protein	Phage protein
ORF31	14736	16748	2013	Forward	CP000114	100.00%	Prophage LambdaSa03, pblA protein, internal deletion	Phage minor tail protein
ORF32	16742	18274	1533	Forward	LT714196	84.87%	Prophage LambdaSa03, tail component	Putative minor structural protein
ORF33	18275	22396	4122	Forward	CP000114	100.00%	Prophage LambdaSa03, minor structural protein, putative	Phage tail fibers
ORF34	22397	24409	2013	Forward	LT714196	97.47%	Prophage LambdaSa03, minor structural protein	Structural protein
ORF35	24421	24813	393	Forward	LT714196	93.89%	Hypothetical protein	Hypothetical protein
ORF36	24836	24964	129	Forward	CP000114	100.00%	Conserved hypothetical protein	
	24764	24964	201	Forward				Hypothetical protein
ORF37	24973	25275	303	Forward	LT714196	100.00%	Phage protein	Phage protein (ACLAME 358)
ORF38	25268	25495	228	Forward	LT714196, NC_004589, DQ864624, NC_004584, NC_009819, NC_004585, AP012335	92.98% – 88.16%	Prophage LambdaSa03, holin; putative holin; holin	Phage holin
ORF39	25504	25620	117	Forward	JX409894, LT714196, JX409895	99.15% – 94.02%	Hypothetical protein	Hypothetical protein
ORF40	25621	26952	1332	Forward	LT714196, JX409895, JX409894	99.02% – 94.22%	Phage lysin, N-acetylmuramoyl-L-alanine amidase; putative lysin	Phage lysin, N-acetylmuramoyl-L-alanine amidase
ORF41	27214	27384	171	Reverse	CP021869, LT714196	100.00%	Hypothetical protein	FIG01116037: hypothetical protein
ORF42	27482	27766	285	Forward				Hypothetical protein
ORF43	27803	27982	180	Forward	CP021869, LT714196	100.00%	Paratox	Paratox
ORF44	28279	29421	1143	Reverse	CP021869, LT714196	96.68%	Prophage LambdaSa03, site-specific recombinase phage integrase; site-specific integrase	Phage integrase
ORF45	29549	29758	210	Reverse	CP000114	100.00%	Conserved hypothetical protein	

ORF46	29810	30190	381	Reverse	JX409894, LT714196	99.74% – 95.80%	Putative CI-like repressor; hypothetical protein	Phage protein
ORF47	30177	30536	360	Reverse	CP000114	100.00%	Prophage LambdaSa03, transcriptional regulator, Cro/CI family	Phage cI repressor (ACLAME 5)
ORF48	30728	30946	219	Forward	KT388093, KY349816, NC_004588, NC_028697	81.28%	Cro-like transcriptional regulator; cro family anti-repressor; putative Cro-like repressor	Transcriptional regulator
ORF49	31044	31376	333	Forward	JX409895	100.00%	Hypothetical protein	Phage protein
ORF50	31481	31816	336	Reverse	LT714196	98.21%	Hypothetical protein	Hypothetical protein
ORF51	31962	32147	186	Forward	JX409895, JX409894	98.39% – 95.70%	Putative DNA-binding phage protein	Putative DNA-binding phage protein
ORF52	32226	32537	312	Forward	CP021869, LT714196	94.23%	Excisionase; putative excisionase	Phage excisionase
ORF53	32542	32691	150	Forward	LT714196, LT714196	99.33% – 83.33%	Hypothetical protein	
ORF54	32685	32774	90	Forward				Hypothetical protein
ORF55	32767	32967	201	Forward	JX409895	100.00%	Hypothetical protein	Hypothetical protein
ORF56	32964	33254	291	Forward	CP000114	100.00%	Conserved hypothetical protein	Hypothetical phage protein
ORF57	33241	33924	684	Forward	CP000114	100.00%	Conserved hypothetical protein TIGR01618	ORF46
ORF58	33999	35363	1365	Forward	CP000114	100.00%	Prophage LambdaSa03, helicase, putative	Putative helicase
ORF59	35368	35850	483	Forward	CP000114	100.00%	Conserved hypothetical protein	Phage protein
ORF60	35868	37421	1554	Forward	CP000114	100.00%	Conserved hypothetical protein/bacteriophage resistance protein	Phage protein
